# Differences in Plasma Lactoferrin Concentrations Between Subjects with Normal Cognitive Function and Mild Cognitive Impairment: An Observational Study

**DOI:** 10.3390/healthcare13080872

**Published:** 2025-04-11

**Authors:** Małgorzata Jamka, Aleksandra Makarewicz-Bukowska, Joanna Popek, Patrycja Krzyżanowska-Jankowska, Hanna Wielińska-Wiśniewska, Anna Miśkiewicz-Chotnicka, Szymon Kurek, Jarosław Walkowiak

**Affiliations:** Department of Pediatric Gastroenterology and Metabolic Diseases, Poznan University of Medical Sciences, Szpitalna Str. 27/33, 60-572 Poznań, Poland; amakarewicz@ump.edu.pl (A.M.-B.); j.popek@ump.edu.pl (J.P.); pkrzyzanowska@ump.edu.pl (P.K.-J.); hwielinska@ump.edu.pl (H.W.-W.); chotnicka@ump.edu.pl (A.M.-C.); skurek@ump.edu.pl (S.K.); jarwalk@ump.edu.pl (J.W.)

**Keywords:** lactoferrin, cognition, cognitive function, Montreal Cognitive Assessment scale

## Abstract

Background: Previous studies suggested that decreased saliva lactoferrin (LF) levels might be used to differentiate subjects with mild cognitive impairment (MCI) from subjects with normal cognitive function (NCF). Here, we aimed to assess differences in plasma LF concentrations between subjects with NCF and MCI. Methods: In total, 113 NCF subjects and 113 MCI individuals were included in this study. Cognitive function was assessed using the Montreal Cognitive Assessment (MoCA) scale, and anthropometric parameters, body composition, physical activity, cardio-metabolic parameters, and LF levels were measured. Results: MCI subjects had significantly lower LF levels than NCF participants (*p* < 0.0001). There were also significant differences between the study groups in the smoking history (*p* = 0.0190), alcohol consumption (*p* = 0.0036), intake of hypoglycaemic drugs (*p* = 0.0140), vigorous activity (MET-min/day: *p* = 0.0223, min/day: *p* = 0.0133), and energy expenditure associated with activity (*p* = 0.0287). Moreover, the MoCA test results significantly correlated with LF levels (*p* = 0.0026), and there were significant differences between MoCA tertiles and LF levels (*p* = 0.0189). Also, adjusted logistic regression analysis results showed that LF concentrations (*p* = 0.0382), alcohol consumption (*p* = 0.0203), and intake of hypoglycaemic drugs (*p* = 0.0455) were independent predictors of MCI prevalence. Conclusions: In conclusion, MCI subjects are characterised by lower plasma LF concentrations than NCF individuals, but further studies are needed to confirm these findings.

## 1. Introduction

The global demographic is shifting, with the elderly population increasing significantly. According to the World Health Organization (WHO), people aged 60 years or older reached approximately one billion in 2019. Projections indicate this number will rise to 1.4 billion by 2030 and 2.1 billion by 2050. This trend towards an ageing population correlates with a higher incidence of various diseases, including cognitive disorders [1]. Among these, mild cognitive impairment (MCI) is particularly common, serving as an intermediate stage between typical cognitive functions and dementia [2]. MCI affects over 15% of individuals aged 50 years and older [3], with its prevalence increasing with age, decreasing with higher education levels [4], and being more common in men [3]. In Poland, the incidence of MCI in the elderly population (≥60 years of age) is similar to its prevalence worldwide. According to the results of the PolSenior 2 study, suspected MCI was identified in 16.8% of seniors, while dementia was found in 15.8% [5]. Importantly, MCI significantly elevates the risk of developing dementia [6] and Alzheimer’s disease (AD) [7], with approximately 15% of MCI subjects progressing to dementia within two years [2]. Therefore, early diagnosis of MCI is crucial for effective management and intervention.

Lactoferrin (LF), a member of the transferrin family of iron-binding proteins, plays a crucial role in many physiological functions due to its widespread distribution in various tissues. This 80 kDa glycoprotein, consisting of 703 amino acids and multiple sialic acid residues [8,9], has been identified in activated microglial cells in some brain regions [10], suggesting its potential neuroprotective properties. In addition, exogenous LF’s ability to cross the blood–brain barrier, facilitated by LF receptors on the membrane of vascular endothelial cells, further supports its therapeutic potential [11,12]. Notably, Mohamed et al. [13] demonstrated that three-month LF administrations in AD patients decreased the levels of acetylcholine, serotonin, antioxidant and anti-inflammatory markers, and β-amyloid 1–42, suggesting LF’s role in AD prevention through the modulation of the phosphorylated protein kinase B/phosphatase and tensin homolog pathway. Moreover, recent studies [14,15] indicated that decreased salivary LF levels can distinguish subjects with cognitive impairment from those with normal cognitive function (NCF). As blood LF levels were not measured in these studies, we aimed to explore differences in plasma LF concentrations between subjects with NCF and MCI. We hypothesised that LF levels do not significantly differ between these two groups.

## 2. Materials and Methods

### 2.1. Ethics

The observational study was performed in accordance with the guidelines of the Declaration of Helsinki [16] and received ethical approval from the Bioethical Committee of the Poznan University of Medical Sciences (protocol code: 47/20 with amendments) on 16 January 2020. Subjects were provided with study information and informed that involvement was voluntary, with the option to withdraw at any time without the need to provide reasons. All participants provided written informed consent. The publication adheres to the guidelines outlined in the Strengthening the Reporting of Observational Studies in Epidemiology (STROBE; see Supplementary Materials Table S1) [17].

### 2.2. Study Population

The participants were enrolled from July 2021 and November 2024 from clinics via advertisements and posters, as well as senior clubs, universities, and corporations in Poznań. Following initial telephone contact, potential participants were screened by a physician at the Department of Pediatric Gastroenterology and Metabolic Diseases to ensure adherence to protocol requirements as described earlier [18]. During this phase, MoCA, the Hamilton depression rating scale (HAM-D), and medical examination were performed, and a medical history questionnaire was compiled. The inclusion criteria were: 50–70 years, MoCA test results: 19–26 points (MCI group) and 27–30 points (NCF group), and residency within the community. The exclusion criteria included: treated depression and/or the HAM-D test results > 13 points, use of cognitive boosting medications or psychotropic medications, alcohol (consumption > 15 units/week) and other substance abuse disorders, psychiatric conditions, Parkinson’s disease, AD, dementia, anaemia, diabetes for at least ten years, severe chronic renal and hepatic diseases, a history of cancer diseases treated chemo- or radiotherapy in the last five years, history of stroke, history of seizures in the past two years, head injury resulting in loss of consciousness and/or immediate post-injury confusion, hypothyroidism with current abnormal thyrotrophic hormone levels, any other chronic severe illnesses which prevent participation in the study, presence of an implanted pacemaker, neurostimulator, high level of physical activity, and other metal components including prosthetic implants, blindness, deafness, communication difficulties, or any other disability that could impede participation or compliance with the protocol. The anthropometric parameters, body composition, physical activity, blood pressure (BP), and biochemical parameters were assessed in all participants included in the study. Moreover, subjects completed a sociodemographic questionnaire. All parameters were evaluated at the Department of Pediatric Gastroenterology and Metabolic Diseases, Poznan University of Medical Sciences, Poznań, Poland.

**Figure 1:**
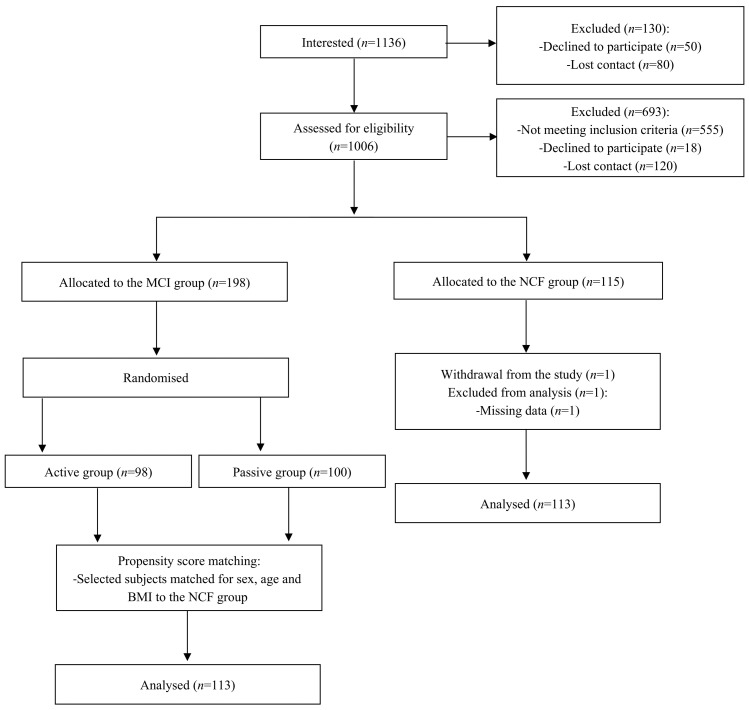
Study flow chart.

In total, 1136 subjects expressed interest in participating in the study, of which 1006 subjects were assessed for eligibility, and 693 individuals were excluded due to not meeting inclusion criteria (*n* = 555), declined to participate in the study (*n* = 18) and lost contact (*n* = 120). The final NCF and MCI groups contained 115 and 198 subjects, respectively. Among 115 subjects assigned to the NCF group, one participant dropped out, and one subjects were excluded from the analysis due to missing data. Out of 198 subjects with MCI recruited for the randomised trial [18], 113 individuals matched for sex, age, and BMI to the NCF group were included in this study. The group of 113 MCI subjects selected for this study did not significantly differ from the total group of 198 MCI individuals. The study flow chart is presented in Figure 1.

### 2.3. Montreal Cognitive Assessment Scale

The MoCA scale was used to detect subtle signs of cognitive impairment. It is a brief 30-item questionnaire that can be completed in 10 min and assesses visuospatial/executive function, naming, memory, attention, language, abstraction, delayed recall, and orientation. The assessment was performed by a physician who completed training and obtained a certificate to administer and score the MoCA. MoCA scores of 27–30 points indicated NCF, 19–26 points suggested MCI, and <19 points diagnosed dementia [19].

### 2.4. Hamilton Depression Rating Scale

The 17-item HAM-D scale was used during the screening to evaluate the prevalence of depressive symptoms in the participants. The assessment was performed by a physician. Each item scored on a scale of 0 to 2 or 0 to 4 points, with the total scores ranging from 0 to 52 points, where ≥23 indicates very severe depression, 18–22 signifies severe depression, 14–18 indicates moderate depression, 8–13 indicates mild depression, and <7 denotes the absence of depression [20,21].

### 2.5. Medical History Questionnaire

A medical history questionnaire was utilised to evaluate the participants’ health conditions and collect information about the subject’s existing medical conditions, past surgical procedures, and injuries. This questionnaire also ascertained whether subjects were taking any medications or dietary supplements.

### 2.6. Anthropometric Parameters

Basic anthropometric markers, including body weight and height and waist and hip circumferences, were evaluated. Body weight and height were measured by an electronic scale with a stadiometer (Radwag, WPT 100/200 OW, Radom, Poland), and measurements were performed with an accuracy of 0.1 kg and 0.5 cm, respectively. Waist and hip circumferences were assessed directly on exposed skin using standard procedures by a measurement tape (Seca 201, Hamburg, Germany) with an accuracy of 0.5 cm. Anthropometric measurements were taken with participants dressed in lightweight attire and barefoot, and an average of two measurements was recorded. BMI and waist-to-hip ratio (WHR) were calculated based on the measurements. The WHO classification of BMI was used to assess subjects’ nutritional status: malnutrition ≤ 18.5 kg/m^2^, normal weight 18.5–24.9 kg/m^2^, overweight 25–29.9 kg/m^2^ and obese ≥ 30 kg/m^2^ [22]. According to the WHO criteria, WHR > 0.85 for women and >0.9 for men indicated android obesity (abdominal obesity). Waist circumference > 80 cm for women and >94 cm for men was considered abdominal obesity [23].

### 2.7. Body Composition

Body composition analysis, including the measurement of fat mass (FM) and visceral adipose tissue (VAT), was performed by dual-energy X-ray absorptiometry methods using the Hologic Discovery analyser (Bedford, MA, USA) in the Department of Pediatric Gastroenterology and Metabolic Diseases. Participants also wore light clothing and removed all metal objects during the assessment. Calibration was performed every day. The American Council on Exercise recommendation was used to diagnose obesity based on the percentage of FM. FM ≥ 32% and 25% in women and men, respectively, suggested obesity [24].

### 2.8. Physical Activity

Physical activity was assessed using the extended version of the International Physical Activity Questionnaire. The questionnaire consists of the following parts: job-related activities, transportation, housework activity, sports, recreational activities, and sitting activities, and includes 27 questions. The questions assess physical activities undertaken within the last seven days and lasting at least 10 min. Each category of physical activity evaluated in the survey was quantified in terms of minutes per day, along with its corresponding MET value. This was achieved by multiplying the coefficient assigned to each specific activity by the weekly frequency in days and the duration in minutes per day. The overall physical activity level was determined by summing up the durations of total walking and moderate and vigorous physical activities [25]. Additionally, activity-related kilocalories were computed using the subsequent formula: one MET = one kcal/kg body mass/h [26].

### 2.9. Blood Pressure

BP was measured in accordance with the European Society of Hypertension guidelines using an electronic sphygmomanometer (Omron M2, HEM-7121-E, Kyoto, Japan). Measurements were taken on the left arm and were represented by three measurements of the systolic (SBP) and diastolic blood pressure (DBP). According to the European Society of Hypertension criteria, a BP < 120/80 mmHg was considered optimal, normal BP was 120–130 mmHg SBP and 80–85 mmHg DBP, while high-normal BP was 130–139/85–89 mmHg, hypertension ≥ 140/90 mmHg, with stage I hypertension indicated by 140–159/90–99 mmHg, stage II hypertension by160–179/100–109 mmHg, and stage III hypertension ≥ 180/110 mmHg [27].

### 2.10. Biochemical Parameters

Blood samples were obtained from the antecubital vein via standard procedures carried out by licensed staff nurses or laboratory diagnosticians. Participants were instructed to avoid physical exertion before blood collection. Fasting blood samples were taken to measure fasting glucose and insulin levels, total cholesterol (TC), low-density lipoprotein cholesterol (LDL-C), HDL-C, TG and high-sensitivity C-reactive protein (hsCRP) in the commercial laboratory. Moreover, plasma LF levels were measured at the Department of Pediatric Gastroenterology and Metabolic Diseases research laboratory by a commercial kit (BIOXYTECH Lactof EIA reagent set, produced by Oxis Research, Oxis International, located in Beverly Hills, CA, USA) using the ELISA method.

The recommendations from the American Diabetes Association were utilised to evaluate glucose metabolism. Normal fasting glucose was characterised by fasting glucose concentrations between 70 and 99 mg/dL, while impaired fasting glucose was defined as fasting glucose levels ranging from 100 to 125 mg/dL. Diagnosis of diabetes mellitus occurred when fasting glucose levels were ≥126 mg/dL (two abnormal test results are needed) or ≥200 mg/dL (random samples for subjects with hyperglycaemia symptoms) [28]. The normal insulin concentrations were considered to be a value within the range of 2–25 µIU/mL. The homeostatic model assessment of insulin resistance (HOMA-IR) was calculated [29], and ≥1.8 indicated insulin resistance according to Adult Treatment Panel III (ATP III) criteria [30]. According to the updates to the ATP III of the National Cholesterol Education Program, optimal LDL-C levels are <100 mg/dL. Desirable concentrations for HDL-C are >40 mg/dL for men and >50 mg/dL for women. TG levels should not exceed 150 mg/dL, while TC levels should remain <200 mg/dL [31]. According to the Centres for Disease Control and Prevention and the American Heart Association, hsCRP levels might be used to estimate the risk of cardiovascular disease. Low risk is at hsCRP concentrations < 1 mg/L, moderate at 1–3 mg/L, and high at >3 mg/L [32].

### 2.11. Sociodemographic Questionnaire

A sociodemographic questionnaire was employed to gather information on participants’ background, residence, education, familial status, and economic situation. Additionally, participants responded to inquiries related to lifestyle elements, tobacco usage patterns, and alcohol consumption.

### 2.12. Minimum Sample Size Calculation

The G*Power 3.1 software (University of Kiel, Kiel, Germany) was used to determine the minimum required sample size based on expected differences in LF concentrations between study groups. The assumptions used for the calculation were as follows: type I error probability (α) = 0.05, type II error probability (β) = 0.2, effect size (mean difference) = 20%, standard deviation = 35% of the mean, and allocation ratio = 1:1. The minimum sample size required was determined to be 63 subjects per group and considering an anticipated maximum dropout rate of 20%, it was recommended to recruit at least 76 subjects for each group to ensure that the sample size remains adequate.

### 2.13. Statistical Analysis

The PQStat 1.8.4 software (PQStst Software, Poznań/Plewiska, Poland) was used for statistical analyses, with a two-sided *p*-value < 0.05 considered as statistically significant. Propensity score matching was applied to select subjects in the study, matched for sex, age, and BMI. The normality of the variables was assessed using the Shapiro–Wilk test. Descriptive statistics for the study population characteristics were presented as medians and Q1–Q3 due to the non-parametric distribution of the data or as frequencies and percentages. Contingency tables and the Fisher exact test or Pearson’s Chi^2^ test were utilised to examine relationships between categorical variables. The Bonferroni correction was applied for multiple comparisons. Unpaired comparisons between two groups were conducted using the Mann–Whitney U test and the Kruskal–Wallis test, and the Dunn post hoc test was applied for comparing three or more groups. The Jonckheere–Terpstra test and the Cochran–Armitage test were calculated for trend analysis. Spearman coefficient correlations were computed to assess the relationships between the chosen variables. Unadjusted logistic regression analysis was employed to identify independent determinants of MCI. Moreover, to identify independent determinants of LF levels, linear regression was performed. To delve deeper into the investigation, the variables from the unadjusted analysis with a significance level of *p* < 0.1 were subsequently included in an adjusted logistic and linear regression model. Multicollinearity variables were excluded from the model.

## 3. Results

### 3.1. Comparison of Subjects with Normal Cognitive Function and Mild Cognitive Impairment

The anthropometric characteristics of the study population are outlined in Table 1, showing no differences between subjects with NCF and MCI. The sociodemographic characteristics of the study population are provided in Table 2, showing significant differences between study groups in the past smoking history (*p* = 0.0190), alcohol consumption (*p* = 0.0036), and frequency of intake of hypoglycaemic drugs (*p* = 0.0140). Moreover, there were differences between MCI and NCF groups in vigorous physical activity level (metabolic equivalent task (MET)-min/day: *p* = 0.0223, min/day: *p* = 0.0133) and energy expenditure associated with activity (*p* = 0.0287, Table 3). However, no differences were found in all analysed biochemical parameters, except LF levels (Table 4), with MCI subjects having significantly lower LF levels compared to NCF participants (median (25th–75th percentile (Q1–Q3)): 172.8 (127.3–223.2) vs. 224.4 (167.0–294.0) ng/mL, *p* < 0.0001).

### 3.2. Comparison of Study Population According to the Montreal Cognitive Assessment Scale Tertiles

The study population was divided into tertiles according to the Montreal Cognitive Assessment (MoCA) results (Table 5), with significant differences between groups in education levels (*p* = 0.0041), financial situation (*p* = 0.0082), sedentary behaviour (*p* = 0.0109), total physical activity level (MET-min/day: *p* = 0.0304, min/day: *p* = 0.0308), energy expenditure associated with activity (*p* = 0.0170), and LF levels (*p* = 0.0189). The lowest MoCA tertile was characterised by a lower percentage of subjects with higher education levels and a higher percentage of individuals from secondary education levels compared to the II tertile group (*p* = 0.0021). This group was also characterised by a lower percentage of subjects with good or very good financial situations than the second tertile group. Moreover, the II tertile group spent more time on sedentary behaviour (*p* = 0.0085) compared to the highest tertile group, and subjects from the lowest tertile group had significantly lower total physical activity (MET-min/day: *p* = 0.0468, min/day: *p* = 0.0405), energy expenditure associated with activity (*p* = 0.0195), and LF concentrations (*p* = 0.0147) than the individuals from the highest tertile group. Trend tests confirmed a significant association between sedentary behaviour (*p* = 0.0373), total physical activity (MET-min/day: *p* = 0.0163, min/day: *p* = 0.0148), energy expenditure associated with activity (*p* = 0.0070) and LF levels (*p* = 0.0056). Moreover, the trend test showed significant differences between tertiles in alcohol consumption (*p* = 0.0416).

### 3.3. Association of Selected Variables with the Prevalence of Mild Cognitive Impairment

Table 6 shows unadjusted odds ratios (OR) and 95% confidence interval (CI) for the associations between analysed variables and the prevalence of MCI. MCI subjects were more likely to take hypoglycaemic medications (*p* = 0.0208), to have a history of current or past smoking (*p* = 0.0095) and to consume alcohol (*p* = 0.0039). They were more likely to have lower total physical activity levels (*p* = 0.0458) and LF levels (*p* = 0.0113). After covariate adjustment, alcohol consumption (*p* = 0.0203), hypoglycaemic drug intake (*p* = 0.0455) and LF levels (*p* = 0.0382) were the only independent predictors of MCI prevalence (Table 7).

### 3.4. Correlations Between the Montreal Cognitive Assessment Scale Results and Selected Variables

Table 8 presents the correlations between the MoCA results and selected variables in the total population, NCF, and MCI groups. The MoCA results positively correlated with LF levels (rho = 0.1997, *p* = 0.0026) in the total population and inversely correlated (rho = −0.1900, *p* = 0.0437) in the MCI groups. Moreover, in the total population, MoCA test results significantly correlated with energy expenditure associated with activity (rho = 0.1494, *p* = 0.0247). In the NCF group, there was a positive correlation between the MoCA results and high-density lipoprotein cholesterol (HDL-C) levels (rho = 0.2113, *p* = 0.0246) and a negative correlation between the MoCA, sedentary behaviour (rho = −0.2194, *p* = 0.0195) and triglycerides (TG) concentrations (rho = −0.2010, *p* = 0.0328).

### 3.5. Association of Selected Variables with Lactoferrin Levels

The results of the univariate regression analysis (Table 9) showed that hip circumference (*p* = 0.0218), VAT mass (*p* = 0.0310), and study group (*p* = 0.0066) were associated with plasma LF levels. However, models 1, 2, and 4 of the multivariate analysis (Table 10), which included variables from the univariate analysis with a significance level of *p* < 0.1 and excluded multicollinear variables, showed that only the study group (model 1: *p* = 0.0176, model 2: *p* = 0.0215, model 4: *p* = 0.0186) was an independent predictor of LF concentrations. In model 3, both the study group (*p* = 0.0069) and FM (*p* = 0.0364) were independent predictors of LF levels.

### 3.6. Correlations Between Lactoferrin Levels and Selected Variables

Correlations between LF concentrations and selected parameters in the total population are presented in Table 11. Positive correlations were noted with BMI (rho = 0.1628, *p* = 0.0143), waist circumference (rho = 0.1466, *p* = 0.0276), hip circumference (rho = 0.2191, *p* <0.0001), FM (rho = 0.1400, *p* = 0.0353), VAT mass (rho = 0.1879, *p* = 0.0046), insulin levels (rho = 0.1407, *p* = 0.0345), and MOCA scores (rho = 0.1997, *p* = 0.0026).

## 4. Discussion

Herein, we showed that subjects with MCI are characterised by lower plasma LF concentrations compared to subjects with NCF. To our knowledge, this is the first study comparing plasma LF levels between MCI and NCF individuals.

Previously, few studies [14,15,33,34] measured LF levels in saliva and cerebrospinal fluid and compared the results obtained in healthy subjects with those with MCI and AD. Carro et al. [14] demonstrated that salivary LF levels more accurately classified NCF, amnestic MCI (aMCI), and AD subjects than β-amyloid 1–42 and total tau measured in cerebrospinal fluid. In that study, salivary LF levels were lower in subjects with aMCI and AD compared with the control group. Moreover, very high correlations were detected between salivary LF concentrations and neurodegenerative markers (total tau and β-amyloid 1–42). Furthermore, Carro et al. [14], in another cohort, demonstrated that low levels of salivary LF (<7.43 μg/mL) in healthy subjects were a significant risk factor for developing aMCI or AD. In addition, salivary LF could distinguish between prodromal AD, AD, and frontotemporal dementia [15]. Moreover, Antequera et al. [34] also reported lower LF concentrations in the saliva of early-onset and late-onset AD patients compared to healthy controls. In another study [35], salivary LF levels exhibited sensitivity to fluctuations in cortical β-amyloid accumulation and showed associations with the thickening of the middle temporal cortex, heightened uptake of fluorodeoxyglucose in the posterior cingulate cortex, and declined memory performance among asymptomatic older individuals. These findings were confirmed in an animal model of AD by Antequera et al. [36], who also observed a reduction of LF in saliva. Interestingly, there were no changes in total protein secretion in saliva in a mouse model of AD compared with wild-type controls. However, in contrast to these results, Gleerup et al. [33] did not find statistically significant differences in LF levels in cerebrospinal fluid or saliva between healthy controls, MCI, AD, and non-AD subjects. Moreover, the authors showed a nonsignificant trend of higher LF levels in the diseased groups compared to the controls, which was opposite to the previous findings, and no relationships were found between LF levels and tau protein, phosphorylated tau, and β-amyloid 1–42 levels. Notwithstanding, a recent meta-analysis confirmed that salivary LF concentrations might serve as a useful biomarker for AD [37].

The mechanism of reducing plasma LF levels in MCI subjects observed in our study is unknown. However, decreased LF concentrations suggest immune system abnormalities, which are frequently observed in subjects with cognitive impairment [38,39]. In addition, it has been suggested that low salivary LF levels could potentially contribute to oral dysbiosis [35], leading to prolonged infections, increased pro-inflammatory response, compromised blood–brain barrier, and facilitating brain tissue colonisation by periodontal bacteria, ultimately accelerating neuroinflammation associated with AD pathology [40,41].

Investigating the molecular mechanisms by which the LF can affect cognitive function, Abdelhamid et al. [42] divided mice into three groups: one control and two interventions. Intervention groups received diets containing 2% of LF or 0.5% of pepsin-hydrolysed LF for three months. The authors reported that both LF-containing diets effectively lowered β-amyloid 1–40 and 1–42 in the brain by impeding the amyloidogenic processing of β-amyloid protein precursor and diminishing β-site amyloid protein precursor cleaving enzyme 1 levels. Guo et al. [43] also showed that human LF administration in an AD mouse model facilitated the non-amyloidogenic processing of amyloid precursor protein, consequently reducing β-amyloid generation and improving spatial cognitive learning ability. Moreover, He et al. [44] concluded that LF might mitigate cognitive impairment by restraining microglial activation and neuroinflammation mediated by the microbiome-gut-brain axis. In that study, the authors reported that 16-week LF supplementation had a positive effect on the length and curvature of postsynaptic density, suppressed microglia activation and proliferation, reduced levels and expression of pro-inflammatory markers in the hippocampus, enhanced the expression of tight junction proteins, and increased the prevalence of *Bacteroidetes* at phylum and *Roseburia* at genus. Interestingly, antibiotic administration inhibited the beneficial effects of LF, indicating the involvement of gut microbiota in LF action. Ran et al. [45] also reported a positive effect of LF supplementation on an AD mouse model. In their study, LF inhibited the progression of AD primarily due to its anti-inflammatory and antioxidative bioactivity, as well as its positive impact on gut microbiota. Another study [13] conducted in AD patients demonstrated that LF administration for three months significantly improved cognitive function, which is associated with the effect on the protein kinase B/phosphatase and tensin homolog pathway, as well as inflammatory and oxidative stress markers. In contrast, Zhou et al. [46] reported no effect of LF supplementation in mice on cognitive function and proteins contributing to the β-amyloid metabolism, tau phosphorylation, neuro-inflammation, and synaptic plasticity. Therefore, further research is needed to determine the exact mechanism of the effect of LF on cognitive functions.

In addition to LF concentrations, significant differences in the frequency of intake of hypoglycaemic drugs between MCI and NCF participants were also observed in our study. Moreover, the use of hypoglycaemic drugs was also identified as an independent predictor of MCI in a multivariate logistic regression analysis. However, previous studies [47,48] examining the association between hypoglycaemic medication use and cognitive outcomes have provided conflicting results. While some research showed a protective effect [47], others demonstrated an increased risk of cognitive decline [48], and it has been suggested that different types of medications might have different effects [47,48].

We also found that MCI subjects more frequently reported current alcohol consumption and a history of smoking. Moreover, alcohol consumption was identified as a factor associated with the probability of MCI development, as shown by multivariate logistic regression analysis. These findings align with previous research showing that alcohol intake is a risk factor for the development of MCI [49] and its progression to dementia [50]. Smoking has also been identified as a risk factor for cognitive impairment [49], with a history of smoking associated with a faster decline in entorhinal cortex volume [51].

Here, we also found significant differences between the MCI and NCF groups in vigorous activity, with higher levels observed in the NCF group. This group was also characterised by higher energy expenditure associated with activity. Energy expenditure also positively correlated with MoCA test results in the total population. Moreover, total physical activity was also associated with a lower probability of having MCI in uni- but not in multivariate logistic regression analysis. We also showed significant differences in sedentary behaviour between MoCA tertile groups. The II tertile group spent more time on sedentary behaviour than the highest tertile group. Furthermore, in the NCF group, there was a negative correlation between the MoCA results and sedentary behaviour. The lowest tertile group also had lower total physical activity levels and energy expenditure from activity compared to the highest tertile. A low level of physical activity is a well-known risk factor for cognitive decline [52], and our previous study [53] showed that objectively measured total physical activity and moderate activity were significantly higher in NCF participants than in MCI subjects.

Previous studies [3,4] also showed that the prevalence of MCI decreased with education level. Indeed, we found that the lowest MoCA tertile was characterised by a lower percentage of subjects with higher education levels and a higher percentage of individuals from secondary education levels compared to the II tertile group.

A worse financial situation was found in subjects from the I MoCA tertile than from the II tertile. These results can be associated with a decline in financial skills in MCI subjects, as was reported previously [54]. Another study suggested that declining financial capacity might be an early sign of AD [55]. It was also suggested that a poor socioeconomic situation is associated with an increased risk of cognitive impairment and dementia [56,57].

Our results also showed that lipid profile might be associated with cognitive function, as there was a positive correlation between the MoCA results and HDL-C levels and a negative correlation between the MoCA and TG concentrations in the NCF group. Our findings are in line with Ong et al. [58] results, which showed that lower TG concentrations were also linked to slightly improved short-term memory. Furthermore, data from several longitudinal studies have shown a correlation between TG levels in midlife and the risk of cognitive impairment in the elderly [59]. Reynolds et al. [60] also reported that higher HDL-C and lower TG predicted better maintenance of cognitive functions compared to age, while Ancelin et al. [61] suggested that low HDL-C and high TG levels may be risk factors for dementia in elderly men.

Recent evidence [62,63] also demonstrated that obesity is associated with developing cognitive impairment, but we did not observe differences between MCI and NCF subjects in BMI (body mass index), as both study groups were matched for this factor. For the same reason, both groups did not differ in age and sex. Nevertheless, it is well known that male sex and older age are associated with a higher likelihood of developing MCI [3,4].

Our findings from the multivariate regression analysis showed that the study group and FM were associated with LF levels. Moreover, positive correlations were observed between LF concentrations and BMI, waist and hip circumferences, FM, VAT mass, insulin levels, and MOCA scores. These results align with our previous findings, which demonstrated positive correlations between serum LF levels and anthropometric parameters as well as insulin levels in obese women [64].

This is the first study that compared plasma LF levels between NCF and MCI subjects. However, low LF levels in serum were previously noted in AD patients [65]. Our study population was thoroughly characterised, with stringent inclusion and exclusion criteria applied. Moreover, propensity score matching was used to match both groups according to age, sex, and BMI, with subjects with depression excluded since it might affect cognitive functions [66].

Possible limitations of the present study include using only the MoCA test to evaluate cognitive function and not confirming the assessment with other tests. Nevertheless, the MoCA scale is the recommended cognitive screening tool for the diagnosis of MCI, with sensitivity and specificity at the cut-off point of 25/26 of 80–100% and 50–75%, respectively [67]. In addition, MoCA is more sensitive for differentiating subjects with MCI from those with NCF than the Mini-Mental State Examination scale [68]. Furthermore, we did not assess neurodegenerative biomarkers, such as brain-derived neurotrophic factor, tau protein, or β-amyloid. Moreover, it is possible that the detected differences in vigorous physical activity, rather than cognitive function, contributed to the differences in LF levels observed in our study between the MCI and NCF groups, as LF is known to be secreted after high- and moderate-intensity exercise [69]. Another constraint is the enrolment of a higher proportion of women compared to men, which is a commonly observed trend wherein women tend to participate more frequently in research studies [70]. Moreover, our study was conducted on Caucasians aged 50–70 years; therefore, the results are not generalisable to other ethnic groups.

## 5. Conclusions

Plasma LF concentrations are lower in MCI subjects than in NCF individuals, but further larger studies are needed to confirm these findings and to assess whether LF concentrations could serve as a potential screening tool for patients with cognitive impairments. Future research should focus on exploring its potential role in routine clinical practice, assessing its diagnostic accuracy in detecting mild and more severe cognitive decline.

## Figures and Tables

**Table 1:** Comparison of anthropometric parameters between subjects with NCF and MCI.

	Median (Q1–Q3)			*p*
	Total (*n* = 226)	NCF (*n* = 113)	MCI (*n* = 113)	
Age [years]	56 (53–61)	56 (53–61)	56 (52–61)	0.6449
BMI [kg/m^2^]	27.33 (24.14–31.40)	27.51 (23.53–32.88)	27.05 (24.49–30.00)	0.6744
Waist circumference [cm]	93 (84–103)	93 (80–106)	93 (85–100)	0.8980
Hip circumference [cm]	105 (100–113)	105.5 (99–118)	105 (101–112)	0.6157
WHR	0.87 (0.81–0.91)	0.86 (0.80–0.91)	0.87 (0.82–0.91)	0.2827
FM [%]	37.8 (32.2–42.5)	37 (31.1–42.6)	39.1 (33.9–42.1)	0.2470
VAT [g]	632 (410–896)	610 (385–934)	640 (463–809)	0.9675

BMI—body mass index; FM—fat mass; MCI—mild cognitive impairment; NCF—normal cognitive function; Q1–Q3—25th–75th percentile; VAT—visceral adipose tissue; WHR—waist-to-hip ratio

**Table 2:** Comparison of sociodemographic parameters between subjects with NCF and MCI.

		*n* (%)			*p*
		Total (*n* = 226)	NCF (*n* = 113)	MCI (*n* = 113)	
Sex	Women	179 (79.20%)	88 (77.88%)	91 (80.53%)	0.6229
	Men	47 (20.80%)	25 (22.12%)	22 (19.47%)	
Place of residence	Village	47 (20.80%)	21 (18.58%)	26 (23.01%)	0.1521
	City < 50,000 inhabitants	22 (9.73%)	7 (6.20%)	15 (13.27%)	
	City of 50,000–500,000 inhabitants	18 (7.96%)	8 (7.08%)	10 (8.85%)	
	City > 500,000 inhabitants	139 (61.50%)	77 (68.14%)	62 (54.87%)	
Education	Vocational	4 (1.77%)	1 (0.89%)	3 (2.65%)	0.2832
	Secondary	37 (16.37%)	15 (13.27%)	22 (19.47%)	
	High	185 (81.86%)	97 (85.84%)	88 (77.88%)	
Socio-occupational status	Employed	199 (88.05%)	96 (84.96%)	103 (91.15%)	0.3990
	Unemployed	3 (1.33%)	2 (1.77%)	1 (0.88%)	
	Pensioner	24 (10.62%)	15 (12.27%)	9 (7.97%)	
Financial situation	Very good	20 (8.85%)	12 (10.62%)	8 (7.08%)	0.0990
	Good	147 (65.05%)	77 (68.14%)	70 (61.95%)	
	Mediocre	57 (25.22%)	22 (19.47%)	35 (30.97%)	
	Bad	2 (0.88%)	2 (1.77%)	0 (0.00%)	
Current smoking	Yes	25 (11.06%)	8 (7.08%)	17 (15.04%)	0.0563
	No	201 (88.94%)	105 (92.92%)	96 (84.96%)	
Past smoking	Yes	84 (37.17%)	33 (29.20%)	51 (45.13%)	0.0190
	No	142 (62.83%)	80 (70.80%)	62 (54.87%)	
Alcohol consumption	Yes	145 (64.16%)	62 (54.87%)	83 (73.45%)	0.0036
	No	81 (35.84%)	51 (45.13%)	30 (26.55%)	
Antihypertensive drugs	Yes	69 (30.53%)	32 (28.32%)	37 (32.74%)	0.4702
	No	157 (67.47%)	81 (71.68%)	76 (67.26%)	
Hypolipidemic drugs	Yes	27 (11.95%)	13 (11.50%)	14 (12.39%)	0.8375
	No	199 (88.05%)	100 (88.50%)	99 (87.61%)	
Hypoglycaemic drugs	Yes	18 (7.97%)	4 (3.54%)	14 (12.39%)	0.0140
	No	208 (92.03%)	109 (96.46%)	99 (87.61%)	
Hypothyroidism drugs	Yes	36 (15.93%)	23 (20.35%)	13 (11.50%)	0.0691
	No	190 (84.07%)	90 (79.65%)	100 (88.50%)	
Hormone replacement therapy ^1^	Yes	10 (5.59%)	5 (5.68%)	5 (5.49%)	1.0000
	No	169 (94.41%)	83 (94.32%)	86 (94.51%)	

^1^ % of women. MCI—mild cognitive impairment; NCF—normal cognitive function

**Table 3:** Comparison of physical activity between subjects with NCF and MCI.

	Median (Q1–Q3)			*p*
	Total (*n* = 226)	NCF (n = 113)	MCI (n = 113)	
Moderate activity [MET-min/day]	152 (64–312)	167 (69–313)	137 (58–283)	0.5778
Moderate activity [min/day]	46 (20–86)	51 (21–86)	39 (19–86)	0.4921
Vigorous activity [MET-min/day]	0 (0–0)	0 (0–11)	0 (0–0)	0.0223
Vigorous activity [min/day]	0 (0–0)	0 (0–1)	0 (0–0)	0.0133
Sedentary behaviour [min/day]	446 (300–549)	411 (300–514)	463 (343–557)	0.2185
Total physical activity [MET-min/day]	328 (190–537)	356 (218–551)	292 (162–499)	0.0779
Total physical activity [min/day]	95 (56–154)	101 (60–163)	90 (46–143)	0.1097
Energy expenditure associated with activity [kcal/day]	420 (229–681)	490 (285–717)	393 (201–632)	0.0287

MCI—mild cognitive impairment; MET—metabolic equivalent of task; NCF—normal cognitive function; Q1–Q3—25th–75th percentile.

**Table 4:** Comparison of blood pressure and biochemical parameters results between subjects with NCF and MCI.

	Median (Q1–Q3)			*p*
	Total (*n* = 226)	NCF (*n* = 113)	MCI (*n* = 113)	
SBP [mmHg] ^1^	127 (116–141)	127 (115–140)	125 (117–142)	0.8448
DBP [mmHg] ^1^	80 (73–87)	80 (72–86)	80 (73–88)	0.8645
Glucose [mg/dL]	94 (88–101)	94 (88–101)	94 (89–101)	0.5832
Insulin [µIU/mL]	6.4 (4.6–9.3)	6.3 (4.9–10)	6.6 (4.4–9.1)	0.7640
HOMA-IR	1.52 (1.09–2.26)	1.52 (1.11–2.35)	1.55 (1.04–2.16)	0.9780
TC [mg/dL]	214 (189–237)	213 (193–237)	216 (189–237)	0.9619
HDL-C [mg/dL]	57 (47–64)	56 (46–65)	58 (47–64)	0.5735
LDL-C [mg/dL]	133 (113–156)	133 (114–157)	133 (111–153)	0.8843
TG [mg/dL]	101 (74–142)	103 (80–143)	100 (73–138)	0.5758
hsCRP [mg/L]	1.15 (0.58–2.54)	1.15 (0.57–2.55)	1.15 (0.58–2.14)	0.7687
LF [ng/mL]	195.8 (147.2–269.6)	224.4 (167.0–294.0)	172.8 (127.3–223.2)	<0.0001

^1^ Total: *n* = 221; NCF: *n* = 111; MCI: *n* = 110. DBP—diastolic blood pressure; HDL-C—high density lipoprotein cholesterol; HOMA-IR—homeostatic model assessment of insulin resistance; hsCRP—high-sensitivity C reactive protein; LDL—low density lipoprotein cholesterol; LF—lactoferrin; MCI—mild cognitive impairment; NCF—normal cognitive function; Q1–Q3—25th–75th percentile; SBP—systolic blood pressure; TC—total cholesterol; TG—triglycerides.

**Table 5:** Comparison of study population according to MOCA tertiles.

		I MOCA ≤ 25 (*n* = 73)	II MOCA: 26–27 (*n* = 77)	III MOCA ≥ 28 (*n* = 76)	*p*	*p* Trend
		*n* (%)				
Sex	Women	60 (82.19%)	58 (75.32%)	61 (80.26%)	0.5624	0.7811
	Men	13 (17.81%)	19 (24.68%)	15 (19.74%)		
Place of residence	Village	20 (27.40%)	12 (15.58%)	15 (19.74%)	0.6471	-
	City < 50,000 inhabitants	8 (10.96%)	7 (9.09%)	7 (9.21%)		
	City of 50,000–500,000 inhabitants	6 (8.22%)	7 (9.09%)	5 (6.58%)		
	City > 500,000 inhabitants	39 (53.42%)	51 (66.24%)	49 (64.47%)		
Education	Vocational	2 (2.74%)	1 (1.30%)	1 (1.32%)	0.0041 ^1^	-
	Secondary	20 (27.40%)	5 (6.49%)	12 (15.79%)		
	High	51 (69.86%)	71 (92.21%)	63 (82.89%)		
Socio-occupational status	Employed	66 (90.41%)	70 (90.90%)	63 (82.90%)	0.4141	-
	Unemployed	1 (1.37%)	0 (0.00%)	2 (2.63%)		
	Pensioner	6 (8.22%)	7 (9.10%)	11 (14.47%)		
Financial situation	Very good	2 (2.74%)	10 (12.99%)	8 (10.53%)	0.0082 ^2^	-
	Good	43 (58.90%)	52 (67.53%)	52 (68.42%)		
	Mediocre	28 (38.36%)	15 (19.48%)	14 (18.42%)		
	Bad	0 (0.00%)	0 (0.00%)	2 (2.63%)		
Current smoking	Yes	9 (12.33%)	11 (14.29%)	5 (6.58%)	0.2887	0.2578
	No	64 (87.67%)	66 (85.71%)	71 (93.42%)		
Past smoking	Yes	32 (43.84%)	30 (38.96%)	22 (28.95%)	0.1576	0.0594
	No	41 (56.16%)	47 (61.04%)	54 (71.05%)		
Alcohol consumption	Yes	54 (73.97%)	47 (61.04%)	44 (57.90%)	0.0963	0.0416
	No	19 (26.03%)	30 (38.96%)	32 (42.10%)		
Antihypertensive drugs	Yes	25 (34.25%)	19 (24.68%)	25 (32.89%)	0.3828	0.8706
	No	48 (65.75%)	58 (75.32%)	51 (67.11%)		
Hypolipidemic drugs	Yes	9 (12.33%)	10 (12.99%)	8 (10.53%)	0.8891	0.7315
	No	64 (87.67%)	67 (87.01%)	68 (89.47%)		
Hypoglycaemic drugs	Yes	10 (13.70%)	4 (5.19%)	4 (5.26%)	0.0891	0.0590
	No	63 (86.30%)	73 (94.81%)	72 (94.74%)		
Hypothyroidism drugs	Yes	11 (15.07%)	9 (11.69%)	16 (21.05%)	0.2775	0.3113
	No	62 (84.93%)	68 (88.31%)	60 (78.95%)		
Hormone replacementtherapy ^3^	Yes	3 (5.00%)	3 (5.17%)	4 (6.56%)	1.0000	0.7086
	No	57 (95.00%)	55 (94.83%)	57 (93.44%)		
Moderate activity [MET-min/day]		120 (51–270)	158 (74–360)	152 (76–273)	0.4300	0.3302
Moderate activity [min/day]		34 (17–73)	51 (26–94)	48 (26–86)	0.2779	0.1072
Vigorous activity [MET-min/day]		0 (0–0)	0 (0–0)	0 (0–9)	0.1610	0.0585
Vigorous activity [min/day]		0 (0–0)	0 (0–0)	0 (0–1)	0.1568	0.0604
Sedentary behaviour [min/day]		450 (343–540)	480 (369–600)	388 (283–491)	0.0109 ^4^	0.0373
Total physical activity [MET-min/day]		260 (139–464)	355 (221–566)	359 (217–575)	0.0304 ^5^	0.0163
Total physical activity [min/day]		72 (43–131)	101 (59–163)	104 (61–163)	0.0308 ^6^	0.0148
Energy expenditure associated with activity [kcal/day]		343 (188–553)	451 (279–695)	487 (274–721)	0.0170 ^7^	0.0070
Age [years]		57 (53–61)	56 (52–62)	56 (53–60)	0.8874	0.6778
BMI [kg/m^2^]		27.34 (24.86–30.82)	26.70 (24.13–31.14)	27.92 (22.92–32.44)	0.8331	0.9717
Waist circumference [cm]		93 (87–98)	91 (83–104)	93 (80–105)	0.9945	0.8899
Hip circumference [cm]		106 (101–113)	105 (99–113)	106 (100–116)	0.8015	0.7195
WHR		0.87 (0.81–0.91)	0.86 (0.82–0.92)	0.86 (0.79–0.91)	0.4779	0.3307
FM [%]		39.3 (33.9–42.3)	37.2 (32.3–41.7)	36.7 (31.1–42.6)	0.5110	0.3174
VAT [g]		637 (469–809)	632 (420–905)	595 (376–922)	0.9719	0.8605
SBP [mmHg] ^8^		129 (119–144)	125 (117–140)	126 (114–138)	0.4047	0.2210
DBP [mmHg] ^8^		80 (73–90)	79 (73–87)	79 (71–86)	0.5820	0.3339
Glucose [mg/dL]		93 (88–100)	95 (89–101)	93 (88–101)	0.7681	0.7470
Insulin [µIU/mL]		6.9 (5.1–9.5)	6.4 (4.4–8.6)	6.3 (5.1–9.5)	0.6826	0.8284
HOMA-IR		1.60 (1.10–2.27)	1.53 (0.98–2.11)	1.45 (1.11–2.31)	0.7467	0.5969
TC [mg/dL]		209 (185–234)	218 (195–246)	208 (193–229)	0.1829	0.7771
HDL-C [mg/dL]		57 (46–64)	56 (48–64)	56 (48–66)	0.8344	0.5781
LDL-C [mg/dL]		131 (105–153)	136 (118–158)	131 (113–155)	0.2803	0.8393
TG [mg/dL]		97 (73–138)	111 (76–145)	97 (73–129)	0.3606	0.7878
hsCRP [mg/L]		1.17 (0.65–2.76)	1.05 (0.51–1.86)	1.18 (0.56–2.82)	0.6956	0.9501
LF [ng/mL]		183.5 (124.5–244.1)	178.4 (143.8–271.0)	219.0 (178.4–278.2)	0.0189 ^9^	0.0056

^1^ I vs. II: *p* = 0.0021; ^2^ I vs. II: *p* = 0.0212; I vs. III: *p* = 0.0216; ^3^ % of women; ^4^ II vs. III: *p* = 0.0085; ^5^ I vs. III: *p* = 0.0468; ^6^ I vs. III: *p* = 0.0405; ^7^ I vs. III: *p* = 0.0195; ^8^ I: *n* = 70; II: *n* = 76; III: *n* = 75; ^9^ I vs. III: *p* = 0.0147. BMI—body mass index; DBP—diastolic blood pressure; FM—fat mass; HDL-C—high density lipoprotein cholesterol; HOMA-IR—homeostatic model assessment of insulin resistance; hsCRP—high-sensitivity C reactive protein; LDL—low density lipoprotein cholesterol; LF—lactoferrin; MOCA—Montreal cognitive assessment scale; SBP—systolic blood pressure; TC—total cholesterol; TG—triglycerides; VAT—visceral adipose tissue; WHR—waist-to-hip ratio.

**Table 6:** Unadjusted results of logistic regression analysis predicting the probability of MCI.

	OR	95% CI	*p*
Sex ^1^	0.922	0.669–1.273	0.6321
Place of residence ^2^	1.144	0.829–1.580	0.4132
Education ^3^	0.762	0.539–1.076	0.1289
Socio-occupational status ^4^	1.824	0.796–4.179	0.1554
Financial situation ^5^	0.775	0.574–1.047	0.0973
Current or past smoking ^6^	1.438	1.093–1.891	0.0095
Alcohol consumption ^6^	1.509	1.141–1.994	0.0039
Antihypertensive drugs ^6^	1.111	0.836–1.474	0.4704
Hypolipidemic drugs ^6^	1.043	0.698–1.559	0.8375
Hypoglycaemic drugs ^6^	1.963	1.108–3.478	0.0208
Hypothyroidism drugs ^6^	0.713	0.493–1.031	0.0724
Hormone replacement therapy ^6^	1.000	0.530–1.885	1.0000
Total physical activity [min/day]	0.997	0.994–0.999	0.0458
Age [years]	0.990	0.943–1.040	0.6981
BMI [kg/m^2^]	0.975	0.929–1.024	0.3174
Waist circumference [cm]	0.994	0.977–1.012	0.5089
Hip circumference [cm]	0.984	0.960–1.009	0.2155
WHR	1.616	0.097–28.866	0.7380
FM [%]	1.027	0.988–1.067	0.1709
VAT [g]	0.999	0.998–1.000	0.4198
SBP [mmHg]	1.002	0.987–1.016	0.8329
DBP [mmHg]	0.999	0.974–1.024	0.9362
Glucose [mg/dL]	1.008	0.986–1.030	0.4669
Insulin [µIU/mL]	0.990	0.964–1.017	0.4835
HOMA-IR	0.973	0.897–1.055	0.5073
TC [mg/dL]	0.999	0.992–1.006	0.8189
HDL-C [mg/dL]	1.001	0.982–1.021	0.8967
LDL-C [mg/dL]	0.999	0.992–1.007	0.7970
TG [mg/dL]	0.999	0.995–1.004	0.6999
hsCRP [mg/L]	0.992	0.910–1.082	0.8631
LF [ng/mL]	0.996	0.994–0.999	0.0113

^1^ Men (1) vs. women (0); ^2^ Village (1) vs. city (0); ^3^ High (1) vs. other (0); ^4^ Employed (1) vs. unemployed (0); ^5^ Very good + good (1) vs. other (0); ^6^ Yes (1) vs. no (0). BMI—body mass index; CI—confidence interval; DBP—diastolic blood pressure; FM—fat mass; HDL-C—high density lipoprotein cholesterol; HOMA-IR—homeostatic model assessment of insulin resistance; hsCRP—high-sensitivity C reactive protein; LDL—low density lipoprotein cholesterol; LF—lactoferrin; MCI—mild cognitive impairment; OR—odds ratio; SBP—systolic blood pressure; TC—total cholesterol; TG—triglycerides; VAT—visceral adipose tissue; WHR—waist-to-hip ratio.

**Table 7:** Adjusted results of logistic regression analysis predicting the probability of MCI.

	OR	95% CI	*p*
Financial situation ^1^	0.610	0.319–1.166	0.1350
Current or past smoking ^2^	1.731	0.948–3.16	0.0741
Alcohol consumption ^2^	2.031	1.116–3.696	0.0203
Hypoglycaemic drugs ^2^	3.517	1.025–12.062	0.0455
Hypothyroidism drugs ^2^	0.458	0.204–1.031	0.0593
Total physical activity [min/day]	0.997	0.994–1.001	0.1200
LF [ng/mL]	0.997	0.995–0.999	0.0382

^1^ Very good + good (1) vs. other (0); ^2^ Yes (1) vs. no (0). CI—confidence interval; LF—lactoferrin; MCI—mild cognitive impairment; OR—odds ratio.

**Table 8:** Correlations between MOCA test results and selected variables.

	Total (*n* = 226)		NCF (*n* = 113)		MCI (*n* = 113)	
	*rho*	*p*	*rho*	*p*	*rho*	*p*
Age [years]	−0.0023	0.9723	−0.0513	0.5897	−0.0669	0.4814
BMI [kg/m^2^]	0.0032	0.9620	−0.0473	0.6182	−0.0651	0.4934
Waist circumference [cm]	−0.0034	0.9596	−0.0623	0.5121	0.0068	0.9429
Hip circumference [cm]	0.0115	0.8638	−0.0094	0.9209	−0.0598	0.5294
WHR	−0.0590	0.3776	−0.0881	0.3534	0.1066	0.2613
FM [%]	−0.0753	0.2593	0.0342	0.7192	−0.0594	0.5317
VAT [g]	−0.0055	0.9342	−0.0514	0.5888	0.0056	0.9534
SBP [mmHg] ^1^	−0.0746	0.2694	−0.1189	0.2140	−0.1373	0.1525
DBP [mmHg] ^1^	−0.0543	0.4216	−0.1126	0.2393	−0.1568	0.1018
Moderate activity [MET-min/day]	0.0391	0.5585	−0.0364	0.7019	0.0635	0.5037
Moderate activity [min/day]	0.0585	0.3812	−0.0203	0.8307	0.0895	0.3458
Vigorous activity [MET-min/day]	0.1202	0.0714	−0.0337	0.7233	−0.0234	0.8060
Vigorous activity [min/day]	0.1246	0.0616	−0.0337	0.7233	−0.0565	0.5518
Sedentary behaviour [min/day]	−0.1256	0.0594	−0.2194	0.0195	0.0130	0.8911
Total physical activity [MET-min/day]	0.1273	0.0559	0.0006	0.9951	0.1019	0.2830
Total physical activity [min/day]	0.1253	0.0600	0.0153	0.8721	0.1107	0.2433
Energy expenditure associated with activity [kcal/day]	0.1494	0.0247	−0.0181	0.8489	0.1033	0.2762
Glucose [mg/dL]	−0.0275	0.6808	−0.0543	0.5678	0.0765	0.4207
Insulin [µIU/mL]	−0.0139	0.8351	−0.0335	0.7246	−0.0877	0.3556
HOMA-IR	−0.0331	0.6201	−0.0584	0.5392	−0.0757	0.4252
TC [mg/dL]	0.0422	0.5277	−0.0181	0.8494	0.1708	0.0704
HDL-C [mg/dL]	0.0456	0.4947	0.2113	0.0246	0.1061	0.2633
LDL-C [mg/dL]	0.0306	0.6471	−0.0446	0.6389	0.1328	0.1611
TG [mg/dL]	0.0052	0.9374	−0.2010	0.0328	0.0671	0.4803
hsCRP [mg/L]	0.0249	0.7094	0.1052	0.2673	−0.0725	0.4453
LF [ng/mL]	0.1997	0.0026	−0.0622	0.5129	−0.1900	0.0437

^1^ Total: *n* = 221; NCF: *n* = 111; MCI: *n* = 110. BMI—body mass index; DBP—diastolic blood pressure; FM—fat mass; HDL-C—high density lipoprotein cholesterol; HOMA-IR—homeostatic model assessment of insulin resistance; hsCRP—high-sensitivity C reactive protein; LDL—low density lipoprotein cholesterol; LF—lactoferrin; MCI—mild cognitive impairment; MOCA—Montreal cognitive assessment scale; NCF—normal cognitive function; SBP—systolic blood pressure; TC—total cholesterol; TG—triglycerides; VAT—visceral adipose tissue; WHR—waist-to-hip ratio.

**Table 9:** Results of univariate linear regression analysis assessing the relationship between LF levels and selected variables.

	β	SE of β	t	*p*
Sex ^1^	0.0200	0.0668	0.2996	0.7647
Place of residence ^2^	−0.0441	0.0667	−0.6600	0.5099
Education ^3^	0.0830	0.0666	1.2468	0.2137
Socio-occupational status ^4^	0.0152	0.0668	0.2274	0.8203
Financial situation ^5^	0.0549	0.0667	0.8233	0.4112
Current or past smoking ^6^	−0.0434	0.0667	−0.06508	0.5158
Alcohol consumption ^6^	−0.1231	0.0664	−1.8571	0.0646
Antihypertensive drugs ^6^	0.0396	0.0667	0.5925	0.5541
Hypolipidemic drugs ^6^	−0.0214	0.0668	−0.3209	0.7486
Hypoglycaemic drugs ^6^	−0.0776	0.0666	−1.1643	0.2455
Hypothyroidism drugs ^6^	−0.0305	0.0668	−0.4596	0.6481
Hormone replacement therapy ^6^	−0.0166	0.0668	−0.2491	0.8035
Total physical activity [MET-min/day]	0.0256	0.0668	0.3829	0.7021
Total physical activity [min/day]	0.0248	0.0668	0.3711	0.7109
Moderate activity [MET-min/day]	−0.0016	0.0668	−0.0244	0.9806
Moderate activity [min/day]	0.0188	0.0668	0.2810	0.7790
Vigorous activity [MET-min/day]	−0.0087	0.0668	−0.1308	0.8961
Vigorous activity [min/day]	−0.0074	0.0668	−0.1107	0.9119
Sedentary behaviour [min/day]	−0.0055	0.0668	−0.0829	0.9339
Energy expenditure associated with activity [kcal/day]	0.0473	0.0667	0.7096	0.4787
Age [years]	0.0379	0.0668	0.5681	0.5705
BMI [kg/m^2^]	0.1153	0.0664	1.7369	0.0838
Waist circumference [cm]	0.1087	0.0664	1.6371	0.1030
Hip circumference [cm]	0.1525	0.0660	2.3105	0.0218
WHR	0.0308	0.0668	0.4611	0.6452
FM [%]	0.1286	0.0663	1.9412	0.0535
VAT [g]	0.1435	0.0661	2.1709	0.0310
SBP [mmHg]	−0.0194	0.0676	−0.2869	0.7745
DBP [mmHg]	0.0213	0.0675	0.3156	0.7526
Glucose [mg/dL]	0.0210	0.0668	0.3149	0.7531
Insulin [µIU/mL]	−0.0099	0.0668	−0.1490	0.8816
HOMA-IR	−0.0135	0.0668	−0.2022	0.8399
TC [mg/dL]	−0.1212	0.0663	−1.8279	0.0689
HDL-C [mg/dL]	−0.0706	0.0666	−1.0586	0.2909
LDL-C [mg/dL]	−0.0924	0.0665	−1.3887	0.1663
TG [mg/dL]	−0.0515	0.0667	−0.7717	0.4411
hsCRP [mg/L]	0.0305	0.0668	0.4569	0.6482
MOCA [points]	0.1043	0.0665	1.5697	0.1179
Group ^7^	−0.1802	0.0657	−2.7419	0.0066

^1^ Men (1) vs. women (0); ^2^ Village (1) vs. city (0); ^3^ High (1) vs. other (0); ^4^ Employed (1) vs. unemployed (0); ^5^ Very good + good (1) vs. other (0); ^6^ Yes (1) vs. no (0); ^7^ Normal cognitive function (NCF − 0) vs. mild cognitive impairment (MCI − 1). BMI—body mass index; DBP—diastolic blood pressure; FM—fat mass; HDL-C—high density lipoprotein cholesterol; HOMA-IR—homeostatic model assessment of insulin resistance; hsCRP—high-sensitivity C reactive protein; LDL—low density lipoprotein cholesterol; LF—lactoferrin; MOCA—Montreal cognitive assessment scale; SBP—systolic blood pressure; SE—standard error; TC—total cholesterol; TG—triglycerides; VAT—visceral adipose tissue; WHR—waist-to-hip ratio.

**Table 10:** Results of multivariate linear regression analysis assessing the relationship between LF levels and selected variables.

	β	SE of β	*t*	*p*
Model 1				
Alcohol consumption ^1^	−0.0872	0.0665	−1.3123	0.1908
BMI [kg/m^2^]	0.0848	0.0664	1.2774	0.2028
TC [mg/dL]	−0.1054	0.0663	−1.5896	0.1133
Group ^2^	−0.1593	0.0666	−2.3915	0.0176
Model 2				
Alcohol consumption ^1^	−0.0909	0.0662	−1.3738	0.1709
Hip circumference [cm]	0.1276	0.0656	1.9441	0.0531
TC [mg/dL]	−0.1039	0.0656	−1.5878	0.1138
Group ^2^	−0.1536	0.0664	−2.314	0.0215
Model 3				
Alcohol consumption ^1^	−0.0694	0.0665	−1.0435	0.2979
FM [%]	0.1379	0.0655	2.1054	0.0364
TC [mg/dL]	−0.1212	0.0648	−1.8703	0.0627
Group ^2^	0.1812	0.0664	2.7270	0.0069
Model 4				
Alcohol consumption ^1^	−0.0928	0.0663	−1.3997	0.1630
VAT [g]	0.1222	0.0659	1.8559	0.0648
TC [mg/dL]	−0.1011	0.0658	−1.5373	0.1256
Group ^2^	0.1572	0.0663	2.3709	0.0186

^1^ Yes (1) vs. no (0); ^2^ Normal cognitive function (NCF − 0) vs. mild cognitive impairment (MCI − 1). BMI—body mass index; FM—fat mass; LF—lactoferrin; SE—standard error; TC—total cholesterol; VAT—visceral adipose tissue.

**Table 11:** Correlations between LF levels and selected variables in the total population (*n* = 226).

	*rho*	*p*
Age [years]	−0.0313	0.6399
BMI [kg/m^2^]	0.1628	0.0143
Waist circumference [cm]	0.1466	0.0276
Hip circumference [cm]	0.2191	<0.0001
WHR	−0.0199	0.7658
FM [%]	0.1400	0.0353
VAT [g]	0.1879	0.0046
SBP [mmHg] ^1^	0.0151	0.8231
DBP [mmHg] ^1^	−0.0164	0.8085
Moderate activity [MET-min/day]	0.08667	0.3614
Moderate activity [min/day]	0.0131	0.8449
Vigorous activity [MET-min/day]	0.0068	0.9183
Vigorous activity [min/day]	0.1298	0.8462
Sedentary behaviour [min/day]	−0.0329	0.6226
Total physical activity [MET-min/day]	0.0641	0.3372
Total physical activity [min/day]	0.0563	0.3991
Energy expenditure associated with activity [kcal/day]	0.1116	0.0941
Glucose [mg/dL]	0.0344	0.6074
Insulin [µIU/mL]	0.1407	0.0345
HOMA-IR	0.1280	0.0547
TC [mg/dL]	−0.1155	0.0831
HDL-C [mg/dL]	−0.0571	0.3928
LDL-C [mg/dL]	−0.0815	0.2224
TG [mg/dL]	0.0350	0.6010
hsCRP [mg/L]	0.1081	0.1051
MOCA [points]	0.1997	0.0026

^1^ *n* = 221; BMI—body mass index; DBP—diastolic blood pressure; FM—fat mass; HDL-C—high density lipoprotein cholesterol; HOMA-IR—homeostatic model assessment of insulin resistance; hsCRP—high-sensitivity C reactive protein; LDL—low density lipoprotein cholesterol; LF—lactoferrin; MOCA—Montreal cognitive assessment scale; SBP—systolic blood pressure; TC—total cholesterol; TG—triglycerides; VAT—visceral adipose tissue; WHR—waist-to-hip ratio.

## Data Availability

The data presented in this study are available on request from the corresponding author. The data cannot be publicly accessed due to disagreements among the study participants.

## Supplementary Materials

The following supporting information can be downloaded at: https://www.mdpi.com/article/10.3390/healthcare13080872/s1, Table S1: STROBE statement checklist.

## Abbreviations

The following abbreviations are used in this manuscript:

AD	Alzheimer’s disease
aMCI	Amnestic mild cognitive impairment
ATPIII	Adult Treatment Panel III
BMI	Body mass index
BP	Blood pressure
CI	Confidence interval
DBP	Diastolic blood pressure
FM	Fat mass
HAM-D	Hamilton depression rating scale
HDL-C	High-density lipoprotein cholesterol
HOMA-IR	Homeostatic model assessment of insulin resistance
hs-CRP	High-sensitivity C-reactive protein
LDL-C	Low-density lipoprotein cholesterol
LF	Lactoferrin
MCI	Mild cognitive impairment
MET	Metabolic equivalent task
MoCA	Montreal Cognitive Assessment
NCF	Normal cognitive function
OR	Odds ratio
Q1–Q3	25th–75th percentile
SBP	Systolic blood pressure
STROBE	Strengthening the Reporting of Observational Studies in Epidemiology
TC	Total cholesterol
TG	Triglycerides
VAT	Visceral adipose tissue
WHO	World Health Organization
WHR	Waist-to-hip ratio

## References

[1] World Health Organization Ageing Data Portal. https://platform.who.int/data/maternal-newborn-child-adolescent-ageing/ageing-data.

[2] Petersen R.C., Lopez O., Armstrong M.J., Getchius T.S.D., Ganguli M., Gloss D., Gronseth G.S., Marson D., Pringsheim T., Day G.S. (2018). Practice Guideline Update Summary: Mild Cognitive Impairment: Report of the Guideline Development, Dissemination, and Implementation Subcommittee of the American Academy of Neurology. Neurology.

[3] Bai W., Chen P., Cai H., Zhang Q., Su Z., Cheung T., Jackson T., Sha S., Xiang Y.T. (2022). Worldwide Prevalence of Mild Cognitive Impairment among Community Dwellers Aged 50 Years and Older: A Meta-Analysis and Systematic Review of Epidemiology Studies. Age Ageing.

[4] Zhang N.J., Qian Z.D., Zeng Y.B., Gu J.N., Jin Y., Li W., Li W., Jin Y. (2022). Incidence and Risk Factors Associated with Progression to Mild Cognitive Impairment among Middle-Aged and Older Adults. Eur. Rev. Med. Pharmacol. Sci..

[5] Błędowski P., Grodzicki T., Mossakowska M., Zdrojewski T., PolSenior2 (2021). Badanie Poszczególnych Obszarów Stanu Zdrowia Osób Starszych, w Tym Jakości Życia Związanej Ze Zdrowiem [PolSenior2. Study of Specific Areas of the Health Status of Older People, Including Health-Related Quality of Life].

[6] Roberts R.O., Knopman D.S., Mielke M.M., Cha R.H., Pankratz V.S., Christianson T.J.H., Geda Y.E., Boeve B.F., Ivnik R.J., Tangalos E.G. (2014). Higher Risk of Progression to Dementia in Mild Cognitive Impairment Cases Who Revert to Normal. Neurology.

[7] Boyle P.A., Wilson R.S., Aggarwal N.T., Tang Y., Bennett D.A. (2006). Mild Cognitive Impairment: Risk of Alzheimer Disease and Rate of Cognitive Decline. Neurology.

[8] Legrand D., Pierce A., Elass E., Carpentier M., Mariller C., Mazurier J. (2008). Lactoferrin Structure and Functions. Adv. Exp. Med. Biol..

[9] Lönnerdal B., Iyer S. (1995). Lactoferrin: Molecular Structure and Biological Function. Annu. Rev. Nutr..

[10] Fillebeen C., Dexter D., Mitchell V., Benaissa M., Beauvillain J.C., Spik G., Pierce A. (1998). Lactoferrin Is Synthesized by Mouse Brain Tissue and Its Expression Is Enhanced after MPTP Treatment. Adv. Exp. Med. Biol..

[11] Ji B., Maeda J., Higuchi M., Inoue K., Akita H., Harashima H., Suhara T. (2006). Pharmacokinetics and Brain Uptake of Lactoferrin in Rats. Life Sci..

[12] Hu K., Li J., Shen Y., Lu W., Gao X., Zhang Q., Jiang X. (2009). Lactoferrin-Conjugated PEG-PLA Nanoparticles with Improved Brain Delivery: In Vitro and in Vivo Evaluations. J. Control. Release.

[13] Mohamed W.A., Salama R.M., Schaalan M.F. (2019). A Pilot Study on the Effect of Lactoferrin on Alzheimer’s Disease Pathological Sequelae: Impact of the p-Akt/PTEN Pathway. Biomed. Pharmacother..

[14] Carro E., Bartolomé F., Bermejo-Pareja F., Villarejo-Galende A., Molina J.A., Ortiz P., Calero M., Rabano A., Cantero J.L., Orive G. (2017). Early Diagnosis of Mild Cognitive Impairment and Alzheimer’s Disease Based on Salivary Lactoferrin. Alzheimers Dement.

[15] González-Sánchez M., Bartolome F., Antequera D., Puertas-Martín V., González P., Gómez-Grande A., Llamas-Velasco S., Herrero-San Martín A., Pérez-Martínez D., Villarejo-Galende A. (2020). Decreased Salivary Lactoferrin Levels Are Specific to Alzheimer’s Disease. EBioMedicine.

[16] Sawicka-Gutaj N., Gruszczyński D., Guzik P., Mostowska A., Walkowiak J. (2022). Publication Ethics of Human Studies in the Light of the Declaration of Helsinki—A Mini-Review. JMS.

[17] von Elm E., Altman D.G., Egger M., Pocock S.J., Gøtzsche P.C., Vandenbroucke J.P. (2008). The Strengthening the Reporting of Observational Studies in Epidemiology (STROBE) Statement: Guidelines for Reporting Observational Studies. J. Clin. Epidemiol..

[18] Jamka M., Makarewicz A., Wasiewicz-Gajdzis M., Brylak J., Wielińska-Wiśniewska H., Pawlak Z., Nowak J.K., Herzig K.-H., Mądry E., Walkowiak J. (2021). App-Assured Essential Physical Activity for the Prevention of Cognitive Decline: Changing Paradigms in Public Health—A Study Protocol for a Randomised Controlled Trial: A Study Protocol of the PA PROTECT Study. JMS.

[19] Nasreddine Z.S., Phillips N.A., Bédirian V., Charbonneau S., Whitehead V., Collin I., Cummings J.L., Chertkow H. (2005). The Montreal Cognitive Assessment, MoCA: A Brief Screening Tool for Mild Cognitive Impairment. J. Am. Geriatr. Soc..

[20] Hamilton M. (1960). A Rating Scale for Depression. J. Neurol. Neurosurg. Psychiatry.

[21] Rush J., First M., Blacker D. (2008). Handbook of Psychiatric Measures.

[22] World Health Organization A Healthy Lifestyle—WHO Recommendations. https://www.who.int/europe/news-room/fact-sheets/item/a-healthy-lifestyle---who-recommendations.

[23] World Health Organization (2008). Waist Circumference and Waist-Hip Ratio: Report of a WHO Expert Consultation.

[24] American Council on Exercise Percent Body Fat Calculator. https://www.acefitness.org/education-and-resources/lifestyle/tools-calculators/percent-body-fat-calculator/.

[25] Biernat E. (2013). International Physical Activity Questionnaire—Polish Long Version. Pol. J. Sport. Med..

[26] Hills A.P., Mokhtar N., Byrne N.M. (2014). Assessment of Physical Activity and Energy Expenditure: An Overview of Objective Measures. Front. Nutr..

[27] Mancia G., Kreutz R., Brunström M., Burnier M., Grassi G., Januszewicz A., Muiesan M.L., Tsioufis K., Agabiti-Rosei E., Algharably E.A.E. (2023). 2023 ESH Guidelines for the Management of Arterial Hypertension The Task Force for the Management of Arterial Hypertension of the European Society of Hypertension Endorsed by the European Renal Association (ERA) and the International Society of Hypertension (ISH). J. Hypertens.

[28] Elsayed N.A., Aleppo G., Aroda V.R., Bannuru R.R., Brown F.M., Bruemmer D., Collins B.S., Hilliard M.E., Isaacs D., Johnson E.L. (2023). 2. Classification and Diagnosis of Diabetes: Standards of Care in Diabetes-2023. Diabetes Care.

[29] Matthews D.R., Hosker J.P., Rudenski A.S., Naylor B.A., Treacher D.F., Turner R.C. (1985). Homeostasis Model Assessment: Insulin Resistance and β-Cell Function from Fasting Plasma Glucose and Insulin Concentrations in Man. Diabetologia.

[30] Gayoso-Diz P., Otero-González A., Rodriguez-Alvarez M.X., Gude F., García F., De Francisco A., Quintela A.G. (2013). Insulin Resistance (HOMA-IR) Cut-off Values and the Metabolic Syndrome in a General Adult Population: Effect of Gender and Age: EPIRCE Cross-Sectional Study. BMC Endocr. Disord..

[31] National Cholesterol Education Program (NCEP) Expert Panel on Detection, Treatment Evaluation and Treatment of High Blood Cholesterol in Adults (Adult Treatment Panel III) (2002). Third Report of the National Cholesterol Education Program (NCEP) Expert Panel on Detection, Evaluation, and Treatment of High Blood Cholesterol in Adults (Adult Treatment Panel III) Final Report. Circulation.

[32] Myers G.L., Rifai N., Tracy R.P., Roberts W.L., Alexander R.W., Biasucci L.M., Catravas J.D., Cole T.G., Cooper G.R., Khan B.V. (2004). CDC/AHA Workshop on Markers of Inflammation and Cardiovascular Disease: Application to Clinical and Public Health Practice: Report from the Laboratory Science Discussion Group. Circulation.

[33] Gleerup H.S., Jensen C.S., Høgh P., Hasselbalch S.G., Simonsen A.H. (2021). Lactoferrin in Cerebrospinal Fluid and Saliva Is Not a Diagnostic Biomarker for Alzheimer’s Disease in a Mixed Memory Clinic Population. EBioMedicine.

[34] Antequera D., Carrero L., Gonzalez-Sanchez M., Cantero J., Orive G., Municio C., Carro E. (2024). Reduced Salivary Lactoferrin Levels in Early-Onset Alzheimer’s Disease. Aging Dis..

[35] Reseco L., Atienza M., Fernandez-Alvarez M., Carro E., Cantero J.L. (2021). Salivary Lactoferrin Is Associated with Cortical Amyloid-Beta Load, Cortical Integrity, and Memory in Aging. Alzheimers Res. Ther..

[36] Antequera D., Moneo D., Carrero L., Bartolome F., Ferrer I., Proctor G., Carro E. (2021). Salivary Lactoferrin Expression in a Mouse Model of Alzheimer’s Disease. Front. Immunol..

[37] Nijakowski K., Owecki W., Jankowski J., Surdacka A. (2024). Salivary Biomarkers for Alzheimer’s Disease: A Systematic Review with Meta-Analysis. Int. J. Mol. Sci..

[38] Prinz M., Priller J. (2017). The Role of Peripheral Immune Cells in the CNS in Steady State and Disease. Nat. Neurosci..

[39] Bermejo-Pareja F., del Ser T., Valentí M., de la Fuente M., Bartolome F., Carro E. (2020). Salivary Lactoferrin as Biomarker for Alzheimer’s Disease: Brain-Immunity Interactions. Alzheimers Dement..

[40] Kamer A.R., Dasanayake A.P., Craig R.G., Glodzik-Sobanska L., Bry M., De Leon M.J. (2008). Alzheimer’s Disease and Peripheral Infections: The Possible Contribution from Periodontal Infections, Model and Hypothesis. J. Alzheimers Dis..

[41] Sweeney M.D., Zhao Z., Montagne A., Nelson A.R., Zlokovic B.V. (2019). Blood-Brain Barrier: From Physiology to Disease and Back. Physiol. Rev..

[42] Abdelhamid M., Jung C.G., Zhou C., Abdullah M., Nakano M., Wakabayashi H., Abe F., Michikawa M. (2020). Dietary Lactoferrin Supplementation Prevents Memory Impairment and Reduces Amyloid-β Generation in J20 Mice. J. Alzheimers Dis..

[43] Guo C., Yang Z.H., Zhang S., Chai R., Xue H., Zhang Y.H., Li J.Y., Wang Z.Y. (2017). Intranasal Lactoferrin Enhances α-Secretase-Dependent Amyloid Precursor Protein Processing via the ERK1/2-CREB and HIF-1α Pathways in an Alzheimer’s Disease Mouse Model. Neuropsychopharmacology.

[44] He Q., Zhang L.L., Li D., Wu J., Guo Y.X., Fan J., Wu Q., Wang H.P., Wan Z., Xu J.Y. (2023). Lactoferrin Alleviates Western Diet-Induced Cognitive Impairment through the Microbiome-Gut-Brain Axis. Curr. Res. Food Sci..

[45] Ran L., Shi J., Lin Y., Xu C., Han Z., Tian S., Qin X., Li Q., Zhang T., Li H. (2024). Evaluation of the Protective Bioactivity and Molecular Mechanism Verification of Lactoferrin in an Alzheimer’s Mouse Model with Ulcerative Enteritis. J. Dairy. Sci..

[46] Zhou H.H., Wang G., Luo L., Ding W., Xu J.Y., Yu Z., Qin L.Q., Wan Z. (2021). Dietary Lactoferrin Has Differential Effects on Gut Microbiota in Young versus Middle-Aged APPswe/PS1dE9 Transgenic Mice but No Effects on Cognitive Function. Food Nutr. Res..

[47] Herath P.M., Cherbuin N., Eramudugolla R., Anstey K.J. (2016). The Effect of Diabetes Medication on Cognitive Function: Evidence from the PATH through Life Study. Biomed. Res. Int..

[48] Wennberg A.M.V., Hagen C.E., Edwards K., Roberts R.O., Machulda M.M., Knopman D.S., Petersen R.C., Mielke M.M. (2018). Association of Antidiabetic Medication Use, Cognitive Decline, and Risk of Cognitive Impairment in Older People with Type 2 Diabetes: Results from the Population-Based Mayo Clinic Study of Aging. Int. J. Geriatr. Psychiatry.

[49] Jones A., Ali M.U., Kenny M., Mayhew A., Mokashi V., He H., Lin S., Yavari E., Paik K., Subramanian D. (2024). Potentially Modifiable Risk Factors for Dementia and Mild Cognitive Impairment: An Umbrella Review and Meta-Analysis. Dement. Geriatr. Cogn. Disord..

[50] Lao Y., Hou L., Li J., Hui X., Yan P., Yang K. (2021). Association between Alcohol Intake, Mild Cognitive Impairment and Progression to Dementia: A Dose-Response Meta-Analysis. Aging Clin. Exp. Res..

[51] Chen M., Hu C., Dong H., Yan H., Wu P. (2021). A History of Cigarette Smoking Is Associated with Faster Functional Decline and Reduction of Entorhinal Cortex Volume in Mild Cognitive Impairment. Aging.

[52] Sofi F., Valecchi D., Bacci D., Abbate R., Gensini G.F., Casini A., Macchi C. (2011). Physical Activity and Risk of Cognitive Decline: A Meta-Analysis of Prospective Studies. J. Intern. Med..

[53] Makarewicz A., Jamka M., Wasiewicz-Gajdzis M., Bajerska J., Miśkiewicz-Chotnicka A., Kwiecień J., Lisowska A., Gagnon D., Herzig K.H., Mądry E. (2021). Comparison of Subjective and Objective Methods to Measure the Physical Activity of Non-Depressed Middle-Aged Healthy Subjects with Normal Cognitive Function and Mild Cognitive Impairment—A Cross-Sectional Study. Int. J. Env. Res. Public. Health.

[54] Martin R.C., Gerstenecker A., Triebel K.L., Falola M., McPherson T., Cutter G., Marson D.C. (2019). Declining Financial Capacity in Mild Cognitive Impairment: A Six-Year Longitudinal Study. Arch. Clin. Neuropsychol..

[55] Mimmack K.J., Sprague E.H., Amariglio R.E., Vannini P., Marshall G.A. (2024). Longitudinal Evolution of Financial Capacity and Cerebral Tau and Amyloid Burden in Older Adults with Normal Cognition or Mild Cognitive Impairment. J. Prev. Alzheimers Dis..

[56] Zhang J., Feng Y., Zhang X., Wang J., Cheng H., Wang Y., Wang J. (2024). Association of Low Socioeconomic Status with Cognitive Decline among Older Persons in Underdeveloped Areas in China—A Data Analysis of the Gansu Aging Study. BMC Geriatr..

[57] Klee M., Leist A.K., Veldsman M., Ranson J.M., Llewellyn D.J. (2023). Socioeconomic Deprivation, Genetic Risk, and Incident Dementia. Am. J. Prev. Med..

[58] Ong K.L., Morris M.J., McClelland R.L., Hughes T.M., Maniam J., Fitzpatrick A.L., Martin S.S., Luchsinger J.A., Rapp S.R., Hayden K.M. (2018). Relationship of Lipids and Lipid-Lowering Medications with Cognitive Function: The Multi-Ethnic Study of Atherosclerosis. Am. J. Epidemiol..

[59] Power M.C., Rawlings A., Sharrett A.R., Bandeen-Roche K., Coresh J., Ballantyne C.M., Pokharel Y., Michos E.D., Penman A., Alonso A. (2018). Association of Midlife Lipids with 20-Year Cognitive Change: A Cohort Study. Alzheimers Dement..

[60] Reynolds C.A., Gatz M., Prince J.A., Berg S., Pedersen N.L. (2010). Serum Lipid Levels and Cognitive Change in Late Life. J. Am. Geriatr. Soc..

[61] Ancelin M.L., Ripoche E., Dupuy A.M., Barberger-Gateau P., Auriacombe S., Rouaud O., Berr C., Carrière I., Ritchie K. (2013). Sex Differences in the Associations between Lipid Levels and Incident Dementia. J. Alzheimers Dis..

[62] Qu Y., Hu H.Y., Ou Y.N., Shen X.N., Xu W., Wang Z.T., Dong Q., Tan L., Yu J.T. (2020). Association of Body Mass Index with Risk of Cognitive Impairment and Dementia: A Systematic Review and Meta-Analysis of Prospective Studies. Neurosci. Biobehav. Rev..

[63] Hovens I.B., Dalenberg J.R., Small D.M. (2019). A Brief Neuropsychological Battery for Measuring Cognitive Functions Associated with Obesity. Obesity.

[64] Jamka M., Krzyżanowska-Jankowska P., Mądry E., Lisowska A., Bogdański P., Walkowiak J. (2019). No Difference in Lactoferrin Levels between Metabolically Healthy and Unhealthy Obese Women. Nutrients.

[65] Gong L., Sun J., Cong S. (2023). Levels of Iron and Iron-Related Proteins in Alzheimer’s Disease: A Systematic Review and Meta-Analysis. J. Trace Elem. Med. Biol..

[66] Steffens D.C. (2012). Depressive Symptoms and Mild Cognitive Impairment in the Elderly: An Ominous Combination. Biol. Psychiatry.

[67] Lin J.S., O’Connor E., Rossom C., Perdue L.A., Eckstrom E. (2013). Screening for Cognitive Impairment in Older Adults: A Systematic Review for the U.S. Preventive Services Task Force. Ann. Intern. Med..

[68] Roalf D.R., Moberg P.J., Xie S.X., Wolk D.A., Moelter S.T., Arnold S.E. (2013). Comparative Accuracies of Two Common Screening Instruments for Classification of Alzheimer’s Disease, Mild Cognitive Impairment, and Healthy Aging. Alzheimers Dement..

[69] Gillum T., Kuennen M., McKenna Z., Castillo M., Jordan-Patterson A., Bohnert C. (2017). Exercise Increases Lactoferrin, but Decreases Lysozyme in Salivary Granulocytes. Eur. J. Appl. Physiol..

[70] Heerman W.J., Bennett W.L., Kraschnewski J.L., Nauman E., Staiano A.E., Wallston K.A. (2018). Willingness to Participate in Weight-Related Research as Reported by Patients in PCORnet Clinical Data Research Networks. BMC Obes..

