# Supplementary material for: Differences in Plasma Lactoferrin Concentrations Between Subjects with Normal Cognitive Function and Mild Cognitive Impairment: An Observational Study
Source: Healthcare (Basel). 2025 Apr 11;13(8):872. doi: 10.3390/healthcare13080872 (PMC12026691; doi:10.3390/healthcare13080872)
Supplement: Supplementary file 1 [file healthcare-13-00872-s001.zip › healthcare-3465536-supplementary.pdf]

Table S1. STROBE Statement—checklist of items that should be included in reports of observational studies

	Item No.	Recommendation	Page No.	Relevant text from manuscript
**Title and abstract**	1	(*a*) Indicate the study's design with a commonly used term in the title or the abstract	1	Differences in plasma lactoferrin concentrations between subjects with normal cognitive function and mild cognitive impairment: an observational study
		(*b*) Provide in the abstract an informative and balanced summary of what was done and what was found	1	Background: Previous studies suggested that decreased saliva lactoferrin (LF) levels might be used to differentiate subjects with mild cognitive impairment (MCI) from subjects with normal cognitive function (NCF). Here, we aimed to assess differences in plasma LF concentrations between subjects with NCF and MCI. Methods: In total, 113 NCF subjects and 113 MCI individuals were included in this study. Cognitive function was assessed using the Montreal Cognitive Assessment (MoCA) scale, and anthropometric parameters, body composition, physical activity, cardio-metabolic parameters, and LF levels were measured. Results: MCI subjects had significantly lower LF levels than NCF participants (p<0.0001). There were also significant differences between the study groups in the smoking history (p=0.0190), alcohol consumption (p=0.0036), intake of hypoglycaemic drugs (p=0.0140), vigorous activity (MET-min/day: p=0.0223, min/day: p=0.0133) and energy expenditure associated with activity (p=0.0287). Moreover, the MoCA test results significantly correlated with LF levels (p=0.0026), and there were significant differences between MoCA tertiles and LF levels (p=0.0189). Also, adjusted logistic regression analysis results showed that LF concentrations (p=0.0382), alcohol consumption (p=0.0203) and intake of hypoglycaemic drugs (p=0.0455) were independent predictors of MCI prevalence. Conclusions: In conclusion, MCI subjects are characterised by lower plasma LF concentrations than NCF individuals, but further studies are needed to confirm these findings.
**Introduction**				
Background/rationale	2	Explain the scientific background and rationale for the investigation being reported	1-2	The global demographic is shifting, with the elderly population increasing significantly. According to the World Health Organization (WHO), people aged 60 years or older reached approximately one billion in 2019. Projections indicate this number will rise to 1.4 billion by 2030 and 2.1 billion by 2050. This trend towards an ageing population correlates with a higher incidence of various diseases, including cognitive disorders [1]. Among these, mild cognitive impairment (MCI) is particularly common, severing as an intermediate stage between typical cognitive functions and dementia [2]. MCI affects over 15% of individuals aged 50 years and older [3], with its prevalence increasing with age, decreasing with higher education levels [4], and being more common in men [3]. In Poland, the incidence of MCI in the elderly population (≥60 years of age) is similar to its prevalence worldwide. According to the results of the PolSenior 2 study, suspected MCI was identified in 16.8% of

				seniors, while dementia was found in 15.8% [5]. Importantly, MCI significantly elevates the risk of developing dementia [6] and Alzheimer's disease (AD) [7], with approximately 15% of MCI sub-jects progressing to dementia within two years [2]. Therefore, early diagnosis of MCI is crucial for effective management and intervention.<br>Lactoferrin (LF), a member of the transferrin family of iron-binding proteins, plays a crucial role in many physiological functions due to its widespread distribution in various tissues. This 80 kDa glycoprotein, consisting of 703 amino acids and multi-ple sialic acid residues [8,9], has been identified in activated microglial cells in some brain regions [10], suggesting its potential neuroprotective properties. In addition, exogenous LF's ability to cross the blood-brain barrier, facilitated by LF receptors on the membrane of vascular endothelial cells, further supports its therapeutic potential [11,12]. Notably, Mohamed et al. [13] demonstrated that three-month LF administrations in AD patients decreased the levels of acetylcholine, serotonin, antioxidant and anti-inflammatory markers, and β-amyloid 1-42, suggesting LF's role in AD prevention through the modulation of the phosphorylated protein kinase B/phosphatase and tensin homolog pathway. Moreover, recent studies [14,15] indicated that decreased salivary LF levels can distinguish subjects with cognitive impairment from those with normal cognitive function (NCF).
Objectives	3	State specific objectives, including any prespecified hypotheses	2	As blood LF levels were not measured in these studies, we aimed to explore differences in plasma LF concentrations between subjects with NCF and MCI. We hypothesized that LF levels do not significantly differ between these two groups.
**Methods**				
Study design	4	Present key elements of study design early in the paper	2	The observational study was performed in accordance with the guidelines of the Declaration of Helsinki [16] and received ethical approval from the Bioethical Committee of the Poznan University of Medical Sciences (protocol code: 47/20 with amendments). Subjects were provided with study information and informed that involvement was voluntary, with the option to withdraw at any time without the need to provide reasons. All participants provided written informed consent. The publication adheres to the guidelines outlined in the Strengthening the Reporting of Observational Studies in Epidemiology (STROBE; see Supplementary Materials, TableS1) [17].
Setting	5	Describe the setting, locations, and relevant dates, including periods of recruitment, exposure, follow-up, and data collection	2	The participants were enrolled from July 2021 and November 2024 from clinics via advertisements and posters, as well as senior clubs, universities, and corporations in Poznań. Following initial telephone contact, potential participants were screened by a physician at the Department of Pediatric Gastroenterology and Metabolic Diseases to ensure adherence to protocol requirements as described earlier [18].,
Participants	6	(*a*) *Cohort study*—Give the eligibility criteria, and the sources and methods of selection of participants. Describe methods of follow-up	2-3	The inclusion criteria were: 50-70 years, MoCA test results: 19-26 points (MCI group) and 27-30 points (NCF group) and residency within the community. The exclusion criteria included: treated depression and/or the HAM-D test results > 13 points, use of cognitive boosting medications or psychotropic medications, alcohol

		Case-control study—Give the eligibility criteria, and the sources and methods of case ascertainment and control selection. Give the rationale for the choice of cases and controls<br>*Cross-sectional study*—Give the eligibility criteria, and the sources and methods of selection of participants		(consumption > 15 units/week) and other substance abuse disorders, psychiatric conditions, Parkinson's disease, AD, dementia, anaemia, diabetes for at least ten years, severe chronic renal and hepatic diseases, a history of cancer diseases treated chemo- or radiotherapy in the last five years, history of stroke, history of seizures in the past two years, head injury resulting in loss of consciousness and/or immediate post-injury confusion, hypothyroidism with current abnormal thyrotrophic hormone levels, any other chronic severe illnesses which prevent participation in the study, presence of an implanted pacemaker, neurostimulator, high level of physical activity and other metal components, including prosthetic implants, blindness, deafness, communication difficulties or any other disability that could impede participation or compliance with the protocol.
		(*b*) *Cohort study*—For matched studies, give matching criteria and number of exposed and unexposed<br>*Case-control study*—For matched studies, give matching criteria and the number of controls per case	6	Propensity score matching was applied to select subjects to the study, matched for sex, age, and BMI.
Variables	7	Clearly define all outcomes, exposures, predictors, potential confounders, and effect modifiers. Give diagnostic criteria, if applicable	2<br><br>3	During this phase, MoCA, the Hamilton depression rating scale (HAM-D), and medical examination were performed, and a medical history questionnaire was compiled.<br>The anthropometric parameters, body composition, physical activity, blood pressure (BP), and biochemical parameters were assessed in all participants included in the study. Moreover, subjects completed a sociodemographic questionnaire. All parameters were evaluated at the Department of Pediatric Gastroenterology and Metabolic Diseases, Poznan University of Medical Sciences, Poznań, Poland.
Data sources/ measurement	8*	For each variable of interest, give sources of data and details of methods of assessment (measurement). Describe comparability of assessment methods if there is more than one group	3-6	2.3 Montreal Cognitive Assessment Scale<br>The MoCA scale was used to detect subtle signs of cognitive impairment. It is a brief 30-item questionnaire that can be completed in 10 minutes and assesses visuospatial/executive function, naming, memory, attention, language, abstraction, delayed recall, and orientation. The assessment was performed by a physician who completed training and obtained a certificate to administer and score the MoCA. MoCA scores of 27-30 points indicated NCF, 19-26 points suggested MCI and < 19 points diagnosed dementia [19].<br>2.4 Hamilton Depression Rating Scale<br>The 17-item HAM-D scale was used during the screening to evaluate the prevalence of depressive symptoms in the participants. The assessment was performed by a physician. Each item scored on a scale of 0 to 2 or 0 to 4 points, with the total scores ranging from 0 to 52 points, where ≥ 23 indicates very severe depression, 18-22 signifies severe depression, 14-18 indicates moderate depression, 8-13 indicates mild depression, and < 7 denotes the absence of depression [20, 21].<br>2.5 Medical History Questionnaire

A medical history questionnaire was utilized to evaluate the participants' health condition and collected information about the subject's existing medical conditions, past surgical procedures, and injuries. This questionnaire also ascertained whether subjects were taking any medications or dietary supplements.

2.6 Anthropometric Parameters

Basic anthropometric markers, including body weight and height and waist and hip circumferences, were evaluated. Body weight and height were measured by an electronic scale with a stadiometer (Radwag, WPT 100/200 OW, Radom, Poland) and measurements were performed with an accuracy of 0.1 kg and 0.5 cm, respectively. Waist and hip circumferences were assessed directly on exposed skin using standard procedures by a measurement tape (Seca 201, Hamburg, Germany) with an accuracy of 0.5 cm. Anthropometric measurements were taken with participants dressed in lightweight attire and barefoot, and an average of two measurements were recorded. BMI and waist-to-hip ratio (WHR) were calculated based on the measurements. The WHO classification of BMI was used to assess subjects' nutritional status: malnutrition ≤ 18.5 kg/m2, normal weight 18.5-24.9 kg/m2, overweight 25-29.9 kg/m2 and obese ≥ 30 kg/m2 [22]. According to the WHO criteria, WHR > 0.85 for women and > 0.9 for men indicated android obesity (abdominal obesity). Waist circumference > 80 cm for women and > 94 cm for men were considered abdominal obesity [23].

2.7 Body Composition

Body composition analysis, including the measurement of fat mass (FM) and visceral adipose tissue (VAT), was performed by dual-energy X-ray absorptiometry methods using the Hologic Discovery analyser (Bedford, MA, USA) in the Department of Pediatric Gastroenterology and Metabolic Diseases. Participants also wore light clothing and removed all metal objects during the assessment. Calibration was performed every day. The American Council on Exercise recommendation was used to diagnose obesity based on the percentage of FM, FM ≥ 32% and 25% in women and men respectively suggested obesity [24].

2.8 Physical Activity

Physical activity was assessed using the extended version of the International Physical Activity Questionnaire. The questionnaire consists of the following parts: job-related activities, transportation, housework activity, sports, recreational activities, and sitting activities, and includes 27 questions. The questions assess physical activities undertaken within the last seven days and lasting at least 10 minutes. Each category of physical activity evaluated in the survey was quantified in terms of minutes per day, along with its corresponding MET value. This was achieved by multiplying the coefficient assigned to each specific activity by the weekly frequency in days and the duration in minutes per day. The overall physical activity level was determined by summing up the durations of total walking and moderate and vigorous physical activities [25]. Additionally, activity-related

kilocalories were computed using the subsequent formula: one MET = one kcal/kg body mass/h [26].

2.9 Blood Pressure

BP was measured in accordance with the European Society of Hypertension guidelines using an electronic sphygmomanometer (Omron M2, HEM-7121-E, Kyoto, Japan). Measurements were taken on the left arm and were represented by three measurements of the systolic (SBP) and diastolic blood pressure (DBP). According to the European Society of Hypertension criteria, a BP < 120/80 mmHg was considered optimal, normal BP was 120-130 mmHg SBP and 80-85 mmHg DBP, while high-normal BP was 130-139/85-89 mmHg, hypertension ≥ 140/90 mmHg, with stage I hypertension indicated by 140-159/90-99 mmHg, stage II hypertension by160-179/100-109 mmHg, and stage III hypertension ≥ 180/110 mmHg [27].

2.10 Biochemical Parameters

Blood samples were obtained from the antecubital vein via standard procedures carried out by licensed staff nurses or laboratory diagnosticians. Participants were instructed to avoid physical exertion before blood collection. Fasting blood samples were taken to measure fasting glucose and insulin levels, total cholesterol (TC), low-density lipoprotein cholesterol (LDL-C), HDL-C, TG and high-sensitivity C-reactive protein (hsCRP) in the commercial laboratory. Moreover, plasma LF levels were measured at the Department of Pediatric Gastroenterology and Metabolic Diseases research laboratory by a commercial kit (BIOXYTECH Lactof EIA reagent set, produced by Oxis Research, Oxis International, located in Beverly Hills, CA, USA) using ELISA method.

The recommendations from the American Diabetes Association were utilised to evaluate glucose metabolism. Normal fasting glucose was characterised by fasting glucose concentrations between 70 and 99 mg/dl, while impaired fasting glucose was defined as fasting glucose levels ranging from 100 to 125 mg/dl. Diagnosis of diabetes mellitus occurred when fasting glucose levels were ≥126 mg/dl (two abnormal test results are needed) or ≥ 200 mg/dl (random samples for subjects with hyperglycaemia symptoms) [28]. The normal insulin concentrations were considered to be a value within the range of 2-25 µIU/ml. The homeostatic model assessment of insulin resistance (HOMA-IR) was calculated [29], and ≥ 1.8 indicated insulin resistance according to Adult Treatment Panel III (ATP III) criteria [30]. According to the updates to the ATP III of the National Cholesterol Education Program, optimal LDL-C levels are < 100 mg/dl. Desirable concentrations for HDL-C are > 40 mg/dl for men and > 50 mg/dl for women. TG levels should not exceed 150 mg/dl, while TC levels should remain < 200 mg/dl [31]. According to the Centres for Disease Control and Prevention and the American Heart Association, hsCRP levels might be used to estimate the risk of cardiovascular disease. Low risk is at hsCRP concentrations < 1 mg/l, moderate at 1-3 mg/l, and high at > 3 mg/l [32].

				2.11 Sociodemographic Questionnaire<br>A sociodemographic questionnaire was employed to gather information on participants' background, residence, education, familial status, and economic situation. Additionally, participants responded to inquiries related to lifestyle elements, tobacco usage patterns, and alcohol consumption.
Bias	9	Describe any efforts to address potential sources of bias	6	Propensity score matching was applied to select subjects to the study, matched for sex, age, and BMI.
Study size	10	Explain how the study size was arrived at	6	The G*Power 3.1 software (University of Kiel, Kiel, Germany) was used to determine the minimum required sample size based on expected differences in LF concentrations between study groups. The assumptions used for the calculation were as follows: type I error probability (α) = 0.05, type II error probability (β) = 0.2, effect size (mean difference) = 20%, standard deviation = 35% of the mean, and allocation ratio = 1:1. The minimum sample size required was determined to be 63 subjects per group and considering an anticipated maximum dropout rate of 20%, it was recommended to recruit at least 76 subjects for each group to ensure that the sample size remains adequate.
Quantitative variables	11	Explain how quantitative variables were handled in the analyses. If applicable, describe which groupings were chosen and why	2<br>8	MOCA test results: 19-26 points (MCI group) and 27-30 points (NCF group).<br>The study population was divided into tertiles according to the MOCA results.
Statistical methods	12	(*a*) Describe all statistical methods, including those used to control for confounding	6	The PQStat 1.8.4 software (PQStst Software Poznań/Plewiska, Poland) was used for statistical analyses, with a two-sided p-value <0.05 considered as statistically significant. Propensity score matching was applied to select subjects to the study, matched for sex, age, and BMI. The normality of the variables was assessed using the Shapiro-Wilk test. Descriptive statistics for the study population characteristics were presented as medians and Q1 – Q3 due to the non-parametric distribution of the data or as frequencies and percentages. Contingency tables and the Fisher exact test or Pearson's Chi2 test were utilised to examine relationships between categorical variables. The Bonferroni correction was applied for multiple comparisons. Unpaired comparisons between two groups were conducted using the Mann-Whitney U test and the Kruskal-Wallis test with the Dunn post-hoc test applied for comparing three or more groups. The Jonckheere-Terpstra test and the Cochran-Armitage test were calculated for trend analysis. Spearman coefficient correlations were computed to assess the relationships between the chosen variables. Unadjusted logistic regression analysis was employed to identify independent determinants of MCI. Moreover, to identify independent determinants of LF levels, linear regression was performed. To delve deeper into the investigation, the variables from the unadjusted analysis with a significance level of p<0.1 were subsequently included in an adjusted logistic and linear regression model. Multicollinearity variables were excluded from the model.
		(*b*) Describe any methods used to examine subgroups and interactions		
		(*c*) Explain how missing data were addressed	3	Among 115 subjects assigned to the NCF group, one participant dropped out, and one subjects were excluded from the analysis due to missing data.

		(*d*) *Cohort study*—If applicable, explain how loss to follow-up was addressed<br>*Case-control study*—If applicable, explain how matching of cases and controls was addressed<br>*Cross-sectional study*—If applicable, describe analytical methods taking account of sampling strategy	3	In total, 1,136 subjects expressed interest in participating in the study, of which 1,006 subjects were assessed for eligibility, and 693 individuals were excluded due to not meeting inclusion criteria (n=555), declined to participate in the study (n=18) and lost contact (n=120). The final NCF and MCI groups contained 115 and 198 subjects, respectively. Among 115 subjects assigned to the NCF group, one participant dropped out, and one subjects were excluded from the analysis due to missing data. Out of 198 subjects with MCI recruited for the randomised trial [53], 113 individuals matched for sex, age, and BMI to the NCF group were included in this study. The study flow chart is presented in Figure 1.
		(*e*) Describe any sensitivity analyses	-	-
**Results**				
Participants	13*	(a) Report numbers of individuals at each stage of study—eg numbers potentially eligible, examined for eligibility, confirmed eligible, included in the study, completing follow-up, and analysed<br>(b) Give reasons for non-participation at each stage	3	In total, 1,136 subjects expressed interest in participating in the study, of which 1,006 subjects were assessed for eligibility, and 693 individuals were excluded due to not meeting inclusion criteria (n=555), declined to participate in the study (n=18) and lost contact (n=120). The final NCF and MCI groups contained 115 and 198 subjects, respectively. Among 115 subjects assigned to the NCF group, one participant dropped out, and one subjects were excluded from the analysis due to missing data. Out of 198 subjects with MCI recruited for the randomised trial [18], 113 individuals matched for sex, age, and BMI to the NCF group were included in this study. The group of 113 MCI subjects selected for this study did not significantly differ from the total group of 198 MCI individuals. The study flow chart is presented in Figure 1.
		(c) Consider use of a flow diagram	3	Figure 1. Study flow chart.
Descriptive data	14*	(a) Give characteristics of study participants (eg demographic, clinical, social) and information on exposures and potential confounders	6	The sociodemographic characteristics of the study population are provided in Table 2, showing significant differences between study groups in the past smoking history (p=0.0190), alcohol consumption (p=0.0036) and frequency of intake of hypoglycaemic drugs (p=0.0140).
		(b) Indicate number of participants with missing data for each variable of interest	7-10	Tables 1-5
		(c) *Cohort study*—Summarise follow-up time (eg, average and total amount)	-	-
Outcome data	15*	*Cohort study*—Report numbers of outcome events or summary measures over time	-	-
		Case-control study—Report numbers in each exposure category, or summary measures of exposure	-	-
		Cross-sectional study—Report numbers of outcome events or summary measures	-	-

Main results	16	(*a*) Give unadjusted estimates and, if applicable, confounder-adjusted estimates and their precision (eg, 95% confidence interval). Make clear which confounders were adjusted for and why they were included	7-11	Tables 1-7
		(*b*) Report category boundaries when continuous variables were categorized	2<br>8	MOCA test results: 19-26 points (MCI group) and 27-30 points (NCF group).<br>The study population was divided into tertiles according to the MOCA results.
		(*c*) If relevant, consider translating estimates of relative risk into absolute risk for a meaningful time period	-	-
Other analyses	17	Report other analyses done—eg analyses of subgroups and interactions, and sensitivity analyses	12-15	Tables 8-11
**Discussion**				
Key results	18	Summarise key results with reference to study objectives	15	Herein, we showed that subjects with MCI are characterised by lower plasma LF concentrations compared to subjects with NCF. To our knowledge, this is the first study comparing plasma LF levels between MCI and NCF individuals.
Limitations	19	Discuss limitations of the study, taking into account sources of potential bias or imprecision. Discuss both direction and magnitude of any potential bias	17-18	Possible limitations of the present study include using only the MoCA test to evaluate cognitive function and not confirming the assessment by other tests. Nevertheless, the MoCA scale is the recommended cognitive screening tool for the diagnosis of MCI with sensitivity and specificity at the cut-off point of 25/26 of 80-100% and 50-75%, respectively [67]. In addition, MoCA is more sensitive for differentiating subjects with MCI from those with NCF than the Mini-Mental State Examination scale [68]. Furthermore, we did not assess neurodegenerative biomarkers, such as brain-derived neurotrophic factor, tau protein, or β-amyloid. Besides, it is possible that the detected differences in vigorous physical activity rather than cognitive function contributed to the differences in LF levels observed in our study between the MCI and NCF groups, as LF is known to be secreted after high- and moderate-intensity exercise [69]. Another constraint is the enrolment of a higher proportion of women compared to men, which is a commonly observed trend wherein women tend to participate more frequently in research studies [70]. Moreover, our study was conducted on Caucasians aged 50-70 years; therefore, the results are not generalisable to other ethnic groups.
Interpretation	20	Give a cautious overall interpretation of results considering objectives, limitations, multiplicity of analyses, results from similar studies, and other relevant evidence	15-17	Previously, few studies [14,15,33,34] measured LF levels in saliva and cerebrospinal fluid and compared the results obtained in healthy subjects with those with MCI and AD. Carro et al. [14] demonstrated that salivary LF levels more accurately classified NCF, amnestic MCI (aMCI), and AD subjects than β-amyloid 1-42 and total tau measured in cerebrospinal fluid. In that study, salivary LF levels were lower in subjects with aMCI and AD compared with the control group. Moreover, very high correlations were detected between salivary LF concentrations and

neurodegenerative markers (total tau and β-amyloid 1-42). Furthermore, Carro et al. [14], in another cohort, demonstrated that low levels of salivary LF (< 7.43 μg/ml) in healthy subjects were a significant risk factor for developing aMCI or AD. In addition, salivary LF could distinguish between prodromal AD, AD, and frontotemporal dementia [15]. Moreover, Antequera et al. [34] also reported lower LF concentrations in the saliva of early-onset and late-onset AD patients compared to healthy controls. In another study [35], salivary LF levels exhibited sensitivity to fluctuations in cortical β-amyloid accumulation and showed associations with the thickening of the middle temporal cortex, heightened uptake of fluorodeoxyglucose in the posterior cingulate cortex, and declined memory performance among asymptomatic older individuals. These findings were confirmed in an animal model of AD by Antequera et al. [36] who also observed a reduction of LF in saliva. Interestingly, there were no changes in total protein secretion in saliva in a mouse model of AD compared with wild-type controls. However, in contrast to these results, Gleerup et al. [33] did not find statistically significant differences in LF levels in cerebrospinal fluid or saliva between healthy controls, MCI, AD, and non-AD subjects. Moreover, the authors showed a nonsignificant trend of higher LF levels in the diseased groups compared to the controls, which was opposite to the previous findings, and no relationships were found between LF levels and tau protein, phosphorylated tau, and β-amyloid 1-42 levels. Notwithstanding, a recent meta-analysis confirmed that salivary LF concentrations might serve as a useful biomarker for AD [37].

The mechanism of reducing plasma LF levels in MCI subjects observed in our study is unknown. However, decreased LF concentrations suggest immune system abnormalities, which are frequently observed in subjects with cognitive impairment [38,39]. In addition, it has been suggested that low salivary LF levels could potentially contribute to oral dysbiosis [35], leading to prolonged infections, increased pro-inflammatory response, compromised blood-brain barrier, and facilitating brain tissue colonization by periodontal bacteria, ultimately accelerating neuroinflammation associated with AD pathology [40,41].

Investigating the molecular mechanisms by which the LF can affect cognitive function, Abdelhamid et al. [42] divided mice into three groups: one control and two interventions. Intervention groups received diets containing 2% of LF or 0.5% of pepsin-hydrolysed LF for three months. The authors reported that both LF-containing diets effectively lowered β-amyloid 1-40 and 1-42 in the brain by impeding the amyloidogenic processing of β-amyloid protein precursor and diminishing β-site amyloid protein precursor cleaving enzyme 1 levels. Guo et al. [43] also showed that human LF administration in an AD mouse model facilitated the non-amyloidogenic processing of amyloid precursor protein, consequently reducing β-amyloid generation and improving spatial cognitive learning ability. Moreover, He et al. [44] concluded that LF might mitigate cognitive impairment by restraining microglial

activation and neuroinflammation mediated by the microbiome-gut-brain axis. In that study, the authors reported that 16-week LF supplementation had a positive effect on the length and curvature of postsynaptic density, suppressed microglia activation and proliferation, reduced levels and expression of pro-inflammatory markers in the hippocampus, enhanced the expression of tight junction proteins, and increased the prevalence of Bacteroidetes at phylum and Roseburia at genus. Interestingly, antibiotic administration inhibited the beneficial effects of LF, indicating the involvement of gut microbiota in LF action. Ran et al. [45] also reported a positive effect of LF supplementation on an AD mouse model. In their study, LF inhibited the progression of AD primarily due to its anti-inflammatory and antioxidative bioactivity, as well as its positive impact on gut microbiota. Another study [13] conducted in AD patients demonstrated that LF administration for three months significantly improved cognitive function, which is associated with the effect on the protein kinase B/phosphatase and tensin homolog pathway, as well as inflammatory and oxidative stress markers. In contrast, Zhou et al. [46] reported no effect of LF supplementation in mice on cognitive function and proteins contributing to the β-amyloid metabolism, tau phosphorylation, neuro-inflammation, and synaptic plasticity. Therefore, further research is needed to determine the exact mechanism of the effect of LF on cognitive functions.

In addition to LF concentrations, significant differences in the frequency of intake of hypoglycaemic drugs between MCI and NCF participants were also observed in our study. Moreover, the use of hypoglycaemic drugs was also identified as an independent predictor of MCI in a multivariate logistic regression analysis. However, previous studies [47,48] examining the association between hypoglycaemic medication use and cognitive outcomes have provided conflicting results. While some research showed a protective effect [47], others demonstrated an increased risk of cognitive decline [48], and it has been suggested that different types of medications might have different effects [47,48].

We also found that MCI subjects more frequently reported current alcohol consumption and a history of smoking. Moreover, alcohol consumption was identified as a factor associated with the probability of MCI development, as shown by multivariate logistic regression analysis. These findings align with previous research showing that alcohol intake is a risk factor for the development of MCI [49] and its progression to dementia [50]. Smoking has also been identified as a risk factor for cognitive impairment [49], with a history of smoking associated with a faster decline in entorhinal cortex volume [51].

Here, we also found significant differences between the MCI and NCF groups in vigorous activity, with higher levels observed in the NCF group. This group was also characterised by higher energy expenditure associated with activity. Energy expenditure also positively correlated with MoCA test results in the total population. Moreover, total physical activity was also associated with a lower probability of

having MCI in uni- but not in multivariate logistic regression analysis. We also showed significant differences in sedentary behaviour between MoCA tertile groups. The II tertile group spent more time on sedentary behaviour than the highest tertile group. Furthermore, in the NCF group, there was a negative correlation between the MoCA results and sedentary behaviour. The lowest tertile group also had lower total physical activity levels and energy expenditure from activity compared to the highest tertile. A low level of physical activity is a well-known risk factor for cognitive decline [52] and our previous study [53] showed that objectively measured total physical activity and moderate activity were significantly higher in NCF participants than in MCI subjects.

Previous studies [3,4] also showed that the prevalence of MCI decreased with education level. Indeed, we found that the lowest MoCA tertile was characterised by a lower percentage of subjects with higher education levels and a higher percentage of individuals from secondary education levels compared to the II tertile group.

A worse financial situation was found in subjects from the I MoCA tertile than the II tertile. These results can be associated with a decline in financial skills in MCI subjects as was reported previously [54] Another study suggested that declining financial capacity might be an early sign of AD [55]. It was also suggested that a poor socioeconomic situation is associated with an increased risk of cognitive impairment and dementia [56,57].

Our results also showed that lipid profile might be associated with cognitive function, as there was a positive correlation between the MoCA results and HDL-C levels and a negative correlation between the MoCA and TG concentrations in the NCF group. Our findings are in line with Ong et al. [58] results, which showed that lower TG concentrations were also linked to slightly improved short-term memory. Furthermore, data from several longitudinal studies have shown a correlation between TG levels in midlife and the risk of cognitive impairment in the elderly [59]. Reynolds et al. [60] also reported that higher HDL-C and lower TG predicted better maintenance of cognitive functions compared to age, while Ancelin et al. [61] suggested that low HDL-C and high TG levels may be risk factors for dementia in elderly men.

Recent evidence [62,63] also demonstrated that obesity is associated with developing cognitive impairment, but we did not observe differences between MCI and NCF subjects in BMI (body mass index) as both study groups were matched for this factor. For the same reason, both groups did not differ in age and sex. Nevertheless, it is well known that male sex and older age are associated with a higher likelihood of developing MCI [3,4].

Our findings form the multivariate regression analysis showed that the study group and FM were associated with LF levels. Moreover, a positive correlations were observed between LF concentrations and BMI, waist and hip circumferences, FM, VAT mass, insulin levels and MOCA scores. These results align with our previous

				findings, which demonstrated positive correlations between serum LF levels and anthropometric parameters as well as insulin levels in obese women [64].<br>This is the first study that compared plasma LF levels between NCF and MCI subjects. However, low LF levels in serum were previously noted in AD patients [65]. Our study population was thoroughly characterised, with stringent inclusion and exclusion criteria applied. Moreover, propensity score matching was used to match both groups according to age, sex, and BMI, with subjects with depression excluded since it might affect cognitive functions [66].
Generalisability	21	Discuss the generalisability (external validity) of the study results	18	Moreover, our study was conducted on Caucasians aged 50-70 years; therefore, the results are not generalisable to other ethnic groups.
**Other information**				
Funding	22	Give the source of funding and the role of the funders for the present study and, if applicable, for the original study on which the present article is based	18	This research was funded by the National Science Centre, grant number UMO-2017/27/B/NZ7/02924. The APC was funded by the Department of Pediatric Gastroenterology and Metabolic Diseases, Poznan University of Medical Sciences.

*Give information separately for cases and controls in case-control studies and, if applicable, for exposed and unexposed groups in cohort and cross-sectional studies.

Note: An Explanation and Elaboration article discusses each checklist item and gives methodological background and published examples of transparent reporting. The STROBE checklist is best used in conjunction with this article (freely available on the Web sites of PLoS Medicine at http://www.plosmedicine.org/, Annals of Internal Medicine at http://www.annals.org/, and Epidemiology at http://www.epidem.com/). Information on the STROBE Initiative is available at www.strobe-statement.org.
